# Chemical Modifications and Design Influence the Potency of *Huntingtin* Anti-Gene Oligonucleotides

**DOI:** 10.1089/nat.2022.0046

**Published:** 2023-03-30

**Authors:** Osama Saher, Eman M. Zaghloul, Tea Umek, Daniel W. Hagey, Negin Mozafari, Mathias B. Danielsen, Alaa S. Gouda, Karin E. Lundin, Per T. Jørgensen, Jesper Wengel, C.I. Edvard Smith, Rula Zain

**Affiliations:** ^1^ Department of Laboratory Medicine, Translational Research Center Karolinska (TRACK), Karolinska Institutet, Karolinska University Hospital, SE-14186 Huddinge, Sweden.; ^2^ Department of Pharmaceutics and Industrial Pharmacy, Faculty of Pharmacy, Cairo University, Cairo, Egypt.; ^3^ Department of Pharmaceutics, Faculty of Pharmacy, Alexandria University, Alexandria, Egypt.; ^4^ Department of Physics, Chemistry and Pharmacy, Biomolecular Nanoscale Engineering Center, University of Southern Denmark, Odense, Denmark.; ^5^ Department of Chemistry, Faculty of Science, Benha University, Benha, Egypt.; ^6^ Centre for Rare Diseases, Department of Clinical Genetics, Karolinska University Hospital, SE-17176 Stockholm, Sweden.

**Keywords:** anti-gene oligonucleotides, Huntington, trinucleotide repeat, fatty acid conjugation, locked nucleic acid, lipofection

## Abstract

Huntington's disease is a neurodegenerative, trinucleotide repeat (TNR) disorder affecting both males and females. It is caused by an abnormal increase in the length of CAG•CTG TNR in exon 1 of the *Huntingtin* gene (*HTT*). The resultant, mutant HTT mRNA and protein cause neuronal toxicity, suggesting that reduction of their levels would constitute a promising therapeutic approach. We previously reported a novel strategy in which chemically modified oligonucleotides (ONs) directly target chromosomal DNA. These anti-gene ONs were able to downregulate both *HTT* mRNA and protein. In this study, various locked nucleic acid (LNA)/DNA mixmer anti-gene ONs were tested to investigate the effects of varying ON length, LNA content, and fatty acid modification on *HTT* expression. Altering the length did not significantly influence the ON potency, while LNA content was critical for activity. Utilization of palmitoyl-modified LNA monomers enhanced the ON activity relatively to the corresponding nonmodified LNA under serum starvation conditions. Furthermore, the number of palmitoylated LNA monomers and their positioning greatly affected ON potency. In addition, we performed RNA sequencing analysis, which showed that the anti-gene ONs affect the “immune system process, mRNA processing, and neurogenesis.” Furthermore, we observed that for repeat containing genes, there is a higher tendency for antisense off-targeting. Taken together, our findings provide an optimized design of anti-gene ONs that could potentially be developed as DNA-targeting therapeutics for this class of TNR-related diseases.

## Introduction

Huntington's disease (HD) is a neurodegenerative disorder with an autosomal dominant inheritance that belongs to the class of trinucleotide repeat (TNR) diseases. The first thorough description of HD was reported in 1872 by the American physician George Huntington [1]. Almost 120 years later, the *Huntingtin* gene (*HTT*) and disease-causing mutation were identified [2]. HD affects both males and females with equal frequency and an estimated prevalence of 5 per 100,000 individuals in North America, Europe, Australia, and parts of Africa [3,4].

HD symptoms combine disturbance of motor, cognitive, and neuropsychiatric functions. The most characteristic features of the disease include dementia and chorea (abnormal involuntary movements) [4]. HD is caused by a CAG•CTG repeat expansion in exon 1 of the *HTT* gene, with full penetrance observed when the number of repeats exceeds 38. The length of the uninterrupted repeats in the mutant allele correlates inversely with disease onset. In addition, these repeats are somatically unstable and expand during a person's lifetime, reaching up to 1,000 in the brain [5]. The consequence of the repeat expansion in the *HTT* gene is the formation of mutant HTT (mHTT) transcript and protein. In most cases, affected individuals do not show any significant symptoms until adulthood. However, extremely long repeat lengths (≥60) in the germ line can lead to the development of a very rare HD form with a juvenile onset.

Clinical diagnosis of HD occurs, on average, at the age of 39. After this, there is a gradual deterioration in health and aggravation of symptoms over a 15–20-year period until death [6].

Although the exact function of *HTT* is not well understood, it is suggested that it contributes to signaling pathways involved in several processes such as autophagy, vesicle transport, and mitosis [7–10]. It is also reported to be critical for neuronal cell survival during development [9,11,12]. The increased length of the glutamine tract in the mutant protein leads to misfolding and aggregation perturbing HTT protein function, resulting in aberrant interactions with other proteins [13–15]. These protein aggregations eventually result in the pathological hallmark of HD observed in patient brains. However, there is also accumulating evidence of *mHTT* mRNA contribution to HD pathogenesis and neurotoxicity [14,16]. The increase in length of the mutant mRNA is predicted to lead to the formation of secondary structures that aberrantly interact and sequester essential proteins inside the cells.

A recent study reported that *mHTT* mRNA-associated toxicity stems from the deregulation of splicing, as most of the sequestered proteins belong to the spliceosome pathway [17].

Unfortunately, there is currently no cure for HD, and the medications prescribed mainly alleviate the symptoms, rather than correcting the underlying neuropathology. Recently, gene therapy strategies targeting RNA and decreasing mHTT protein were investigated. Antisense oligonucleotides (ASOs) with phosphodiester (PO) or phosphorothioate (PS) backbones were reported to successfully downregulate *HTT* [18,19]. Hence, tominersen, an ASO of the gapmer type, targeting *HTT* RNA entered phase 3 clinical trials and showed a sustained reduction in the corresponding mRNA and protein levels [20]. The trial was terminated after recommendation from the independent data monitoring committee because there was no clinical benefit and potential side effects [21,22].

Exploiting the RNA interference machinery to degrade the *mHTT* RNA is another potential strategy [23–26]. Therapeutic approaches utilizing splice switching oligonucleotide (ON) have also been reported. The idea was to promote exon skipping of the caspase 3 and 6 cleavage sites and prevent the formation of toxic *HTT* fragments [27,28]. Successful editing of the *HTT* gene has been reported using zinc finger protein [29] and CRISPR/Cas9 systems [30,31]. While the permanent restoration of gene function is warranted, the challenge is to efficiently deliver these high molecular-weight tools into the affected neuronal cells.

Several chemical modifications are available to enhance the ON efficiency and reduce the toxicity associated with the existing ASO and siRNA therapeutics [32]. One example is to modify the ON with lipophilic moieties. Cholesterol and α-tocopherol were among the first of many that were introduced and proved successful [33]. Recently, a study comparing different lipophilic conjugations to ASOs showed that cholesterol conjugation caused toxicity. The same study reported that α-tocopherol and palmitoyl conjugations have comparable efficiency. However, the effect observed with α-tocopherol-conjugated ASOs was less consistent upon changing the injection route compared with palmitoyl-conjugated ASOs [34]. Palmitoyl-conjugated gapmers have also been reported to increase the circulatory half-life of the ON in circulation by binding to albumin [35,36]. Furthermore, another study reported conjugating palmitic acid to a thio-phosphoramidate anticancer ON that binds and inhibits the telomerase enzyme [37].

**FIG. 1.**
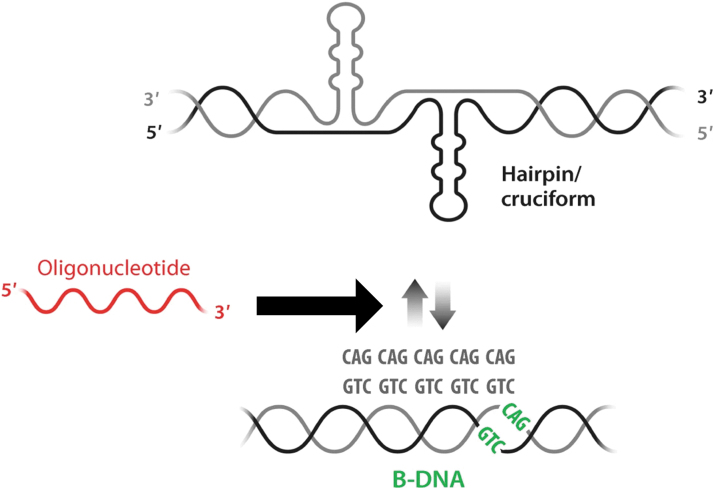
The concept of anti-gene therapeutics in HD (Adapted from Zain and Smith^55^). Expansion of repeat sequences (CAG•CTG) causes the establishment of an equilibrium between B-DNA and non-B-DNA structures (hairpin/cruciform conformation) at the expanded regions. It is hypothesized that the anti-gene CAG ON composed of LNA/DNA mixmer (in *red*) would affect the non-B-DNA structures (hairpin) induced upon the CAG•CTG expansion. HD, Huntington's disease; LNA, locked nucleic acid; ON, oligonucleotide.

We have pioneered a strategy in which ONs bind directly to the *HTT* gene and block its transcription, with resultant effects on both mRNA and protein levels [38,39]. We believe that the interference through binding at the DNA level could potentially cause a sustained downregulation, possibly enabling lower dosing compared with ASOs, as schematically depicted in Fig. 1. We successfully managed to reduce the levels of *HTT* mRNA and protein for up to 10 days in HD patient fibroblasts using locked nucleic acid (LNA)/DNA ON mixmers targeted to CAG•CTG repeats in the template strand of the *HTT* gene. In addition, we observed that our ON treatment significantly impaired RNA polymerase II serine 2 phosphorylation at the *HTT* gene supporting the idea that the effects were manifested at the chromatin level [38].

With the aim of screening various LNA/DNA ON mixmers targeting the *HTT* gene, we herein attempt to correlate length, LNA content, and the number and position of fatty acid-modified LNA units with ON activity. In addition, we tested the potential application of elongated ONs, either in continuous sequences or as multimers connected through linkers. Our findings provide an understanding of structure–activity relationships of anti-gene ONs, which will be helpful for future synthesis and application of optimal designs.

## Materials and Methods

All the LNA-containing ONs were either synthesized at the Nucleic Acid Centre, University of Southern Denmark, or purchased from Eurogentec^®^ (Seraing, Belgium). CAG sequences were used to target the DNA template strand, whereas CTG sequences were designed to target the mRNA and be used for comparison. ON lengths varied from 13-mers up to 45-mers with either PO or PS backbone, unless otherwise mentioned. Palmitoyl-modified ONs were synthesized according to previously described procedures [36]. Several irrelevant or scrambled ON sequences and an siRNA targeting outside the *HTT* repeat sequence (Sigma^®^) were used as negative and positive controls, respectively. All ON sequences used in this study are listed in Table 1.

**Table 1.** Oligonucleotides Used in the Study

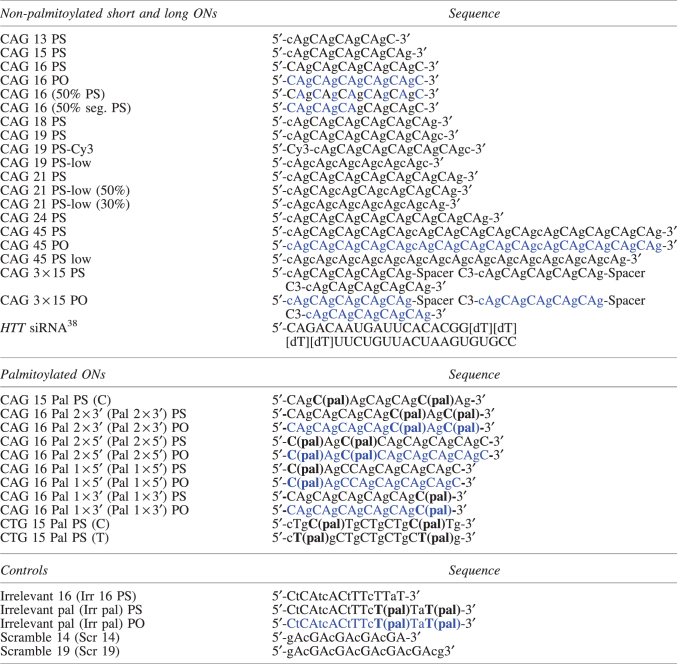

Capital letters: LNA; small letters and dT: DNA. **C(pal)** and **T(pal)**: pal modified 2′-amino-LNA. Black letters represent a PS linkage in the 3′ direction (to the right side), and Blue letters represent a PO linkage in the 3′ direction (to the right side). All single-stranded ONs mentioned have C nucleotides as 5-methyl-C nucleotides.

*HTT, Huntingtin* gene; LNA, locked nucleic acid; ON, oligonucleotide; pal, palmitoyl; PO, phosphodiester; PS, phosphorothioate.

### Cell culture and transfection

GM04281, GM04022, and GM09197 primary fibroblasts from HD patients were obtained from the Coriell Institute for Medical Research, NJ. GM04281 cells carry mutant/wild-type *HTT* alleles with 68/17 CAG•CTG repeats. GM04022 cells carry 44/12 repeats, while GM09197 has 151/21 repeats. Cells were grown in Dulbecco's modified Eagle's medium (DMEM^®^) with l-glutamine, pyruvate, and low glucose (Gibco, Invitrogen, Sweden) supplemented with 10% fetal bovine serum (Invitrogen) and were maintained at 37°C, 5% CO_2_ in humidified incubators.

Transfection was performed as previously described by Zaghloul *et al.* [38]. One day before treatment, cells were seeded at a density of 30,000 cells per well in a 24-well plate or at 120,000 cells per well in a 6-well plate. On treatment day, ONs were formulated with Lipofectamine RNAiMAX^®^ (Invitrogen) according to the manufacturer's protocol using DMEM. Formulations were added to the fibroblasts, while still growing in 10% fetal bovine serum medium, to give a 100 nM final concentration of the ON. Wells assigned as untreated were left untouched until the end of the experiment. Cells were maintained for 4 days before lysis for RNA or protein evaluation.

For serum starvation experiments, we removed the growth media before addition of the complexes (formulated in DMEM). The complexes were incubated with the cells for 4 h followed by replacing the complexes with fresh growth media for another 92 h before lysis and further processing.

### RNA isolation and quantitative reverse transcriptase multiplex polymerase chain reaction

Cells were lysed at assigned time points, and total RNA was isolated using the RNeasy plus kit (Qiagen, Sweden). RNA was analyzed using quantitative reverse transcriptase multiplex polymerase chain reaction (RT-qPCR) (Quantifast^®^ Multiplex RT-PCR kit; Qiagen) to amplify both *HTT* and *HPRT1* (serving as an endogenous control). Sequences of primers and probes for *HTT* and *HPRT1* (Sigma) were as follows: HTT-fwd: 5′-gactcgaacaagcaagag, HTT-rev: 5′-gcctttaacaaaaccttaatttc; HPRT1-fwd: 5′-gagctattgtaatgaccagtc, HPRT1-rev: 5′-tgaccaaggaaagcaaag; HTT TaqMan probe: 5′-[JOE]gaagaatcagtccaggagacc[BHQ1] and HPRT1 TaqMan probe: 5′-[6FAM]tgccagtgtcaattatatcttccacaa[BHQ1]. JOE and 6-FAM represent two fluorophores with different emission spectra and (BHQ1) is a Black Hole Quencher. The multiplex RT-qPCR setup was performed according to the Quantifast kit protocol using 35 ng of RNA for all reactions and a final volume of 25 μL per reaction.

Cycling conditions of PCR were as follows: 20 min at 50°C for reverse transcription, 5 min, 95°C for PCR initial activation step and 45 cycles, each of 2 steps: 15 s, 95°C denaturation and 30 s, 60°C for annealing/extension. Quantitative RT-PCR was performed using the StepOnePlus^®^ Real-time PCR system (Applied Biosystems, Sweden) and the data were analyzed by the ΔΔC_t_ method using the StepOne^®^ software version 2.2.

### Western blotting

Cells were washed, trypsinized, and pelleted. Eppendorf tubes containing pellets were shaken for 1.5 h at 4°C with a RIPA lysis buffer. The lysates were then centrifuged at maximum speed for 10 min after which the supernatants were transferred to other tubes. Five microliter samples were taken from the lysates to quantify the protein using DC Protein Assay (Bio-Rad) according to the manufacturer's protocol. The lysates were mixed with NuPAGE^®^ LDS sample buffer (Invitrogen) in a ratio of 3:1, respectively. Samples were subsequently further processed or stored at −80°C for later analysis. Before loading the gels, 30 μL of the cell lysate (containing ∼30 μg protein) was mixed with 3 μL of NuPAGE sample reducing agent 10× (Invitrogen) and heated at 95°C for 5 min then left to cool down for another 5 min.

Proteins were separated on NuPAGE 3%–8% Tris-acetate gels at 70 V for 30 min followed by 125 V for 6 h on ice or 200 V for 90 min at room temperature. The gels were then transferred using the iBlot 2^®^ Dry Blotting System (Invitrogen) onto nitrocellulose membranes, after which the membranes were blocked with Odyssey Blocking Buffer (LI-COR Biosciences GmbH) for 1 h. Membranes were probed overnight using the anti-HTT primary antibody (ab109115; Abcam) and anti-importin 7 antibody (ab99273; Abcam), used as a housekeeping control. Both antibodies were used at a dilution of 1:1,000. Western blot signals were detected after soaking with secondary antibodies (RDye^®^ 800CW goat anti-rabbit; LI-COR Biotechnology) for 1.5 h, followed by scanning of the membranes using the Odyssey Imager (LI-COR Biosciences GmbH). The signal intensity was measured using function measure on ImageJ after background correction.

### Fluorescence microscopy

Cells were transfected with Cy3-labeled CAG 19 ON to obtain a final concentration of 100 nM. After 4 days, cells were washed twice with phosphate-buffered saline (PBS) after which a nuclear stain (Hoechst 33342; Thermo Fisher Scientific) was added for 15 min followed by washing twice with PBS. Live cell imaging was performed using an Olympus IX81 fluorescence microscope (Olympus America, Inc., Center Valley, PA) while applying an excitation filter 530–550 nm.

### Viability assay

WST-1 reagent (Roche, Germany) was used to assess the viability of cells upon treatment with selected ONs. Cells were seeded in 96-well plates at a density of 9,000 cells/well and transfected the day after. Four days after treatment, the culture medium was replaced with a fresh medium supplemented with the WST-1 reagent (dilution 1:10). The cells were incubated for 2 h at 37°C in a humidified incubator with 5% CO_2_. Absorbance measurements were done at 450 nm with a reference wavelength of 650 nm (SpectraMax i3x; Molecular Devices). Values were expressed as the percentage of the ratio of absorbance at 450 nm of treated cells to the untreated cells.

### RNA sequencing and bioinformatic analysis

The cells were treated for 4 days, after which the RNA was isolated using the RNeasy Kit (Qiagen). The RNA concentration was determined using the Qubit™ RNA High Sensitivity Assay Kit (Invitrogen, Thermo Fisher Scientific) and RNA diluted to 0.8 ng/μL with water [40]. Sequencing was performed in the Bioinformatics and Expression Analysis core facility at Karolinska Insitutet. Sequencing was performed on the same lane and the sequencing depth—900 pM, PE 51 + 51 cycles. The RNAseq library was prepared using the Smart-seq3 sequencing protocol [41]. Fifty base pair paired ends were sequenced on an Illumina HiSeq 2000 sequencer. Bcl files were converted and demultiplexed to FASTQ using the bcl2fastq v2.20.0.422 program. STAR 2.7.5b [42] was used to index the human reference genome (GRCh38) and align the resulting FASTQ files. Mapped reads were then counted in annotated exons using featureCounts v1.5.1 [43].

The gene annotations (Homo_sapiens.GRCh38.101.gtf) and reference genome were obtained from Ensembl. The count table from featureCounts was imported into R/Bioconductor, and differential gene expression was performed using the EdgeR [44] package and its general linear model pipeline. For the gene expression analysis, genes that had 1 count per million in three or more samples were used and normalized using TMM (trimmed mean of the M-values) normalization. Genes with an FDR-adjusted *P* value <0.05 were termed significantly regulated. Plotting was performed in R using the ggplot2 [45] package. CAG repeats were identified genome wide using Homer [46] and its scanMotifGenomeWide.pl function.

Up- and downregulated genes with an adjusted *P* value *<*0.05 were analyzed using Panther Gene Ontology Analysis (panther.org), with a set of all up- and downregulated genes used as a control group. Gene Ontology (GO)-term fold enrichments were then divided by the enrichment in the control group to arrive at the term fold enrichment displayed [47]. Overlap enrichment scores were calculated as follows: (number of genes overlapping between the two sets)/(number of genes in gene set 1× number of genes in gene set 2) [48].

### Data analysis

Data are expressed as mean with standard error of the mean. Statistical significance was determined by one or two-way analysis of variance (ANOVA) followed by individual comparisons using the Bonferroni test (GraphPad Prism 6 Software; GraphPad Software, Inc.). In all cases, *P* < 0.05 was considered significant.

## Results and Discussion

### LNA content, but not length, is critical for LNA/DNA mixmer anti-gene activity

**FIG. 2.**
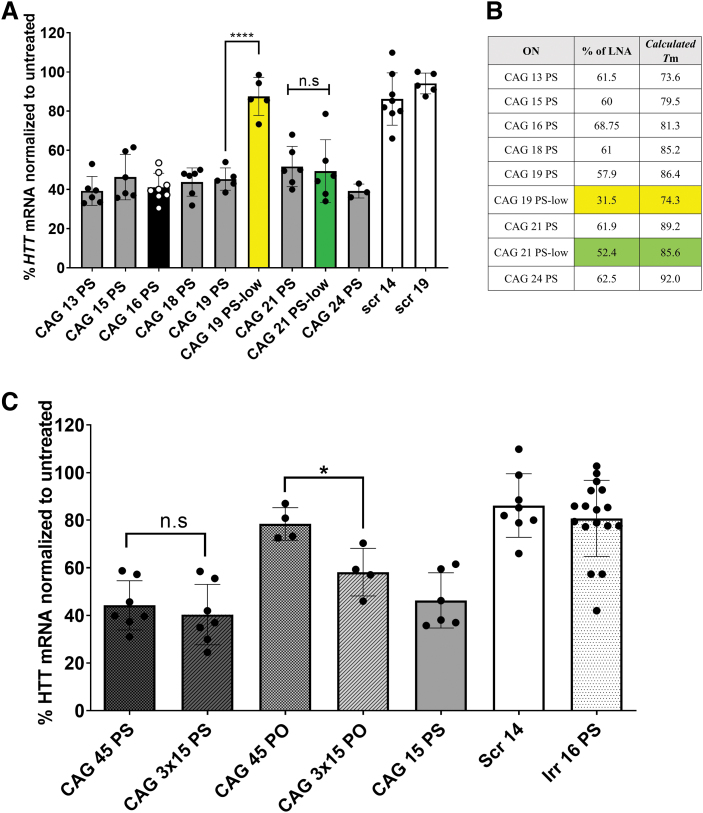
Effect of LNA content and ON lengths on the efficiency of downregulating *HTT* mRNA. **(A)** *HTT* mRNA levels 4 days after transfection of GM04281 human HD fibroblasts carrying 68 repeats on the disease allele with ON (100 nM). **(B)** Percentage of LNA and calculated *T*_m_ in the different ONs used. **(C)** *HTT* mRNA levels 4 days after transfection of GM04281 human HD fibroblasts with long ONs having a different backbone (100 nM). Error bars = SD (*n* ≥ 3), n.s., nonsignificant, *P ≤ 0.05, ****P ≤ 0.0001 (one-way ANOVA, *post hoc* Bonferroni). ANOVA, analysis of variance; *HTT*, *Huntingtin* gene; SD, standard deviation *T*_m_, melting temperature.

Treatment of the GM04281 HD patient fibroblasts with anti-gene ONs, CAG 19, 21, or 24 carrying a fully PS-modified backbone, resulted in 50%–60% downregulation of the *HTT* gene expression comparable with that obtained using shorter ONs, as depicted in Fig. 2A. Bhagat *et al.* [49] emphasized that varying the length of PS ON had a minimal effect on the silencing activity for different mRNA targets. Cytoplasmic and nuclear localizations were observed after transfection with the corresponding Cy3-labeled CAG ON using the same protocol (Supplementary Fig. S1).

However, ONs with a lower LNA content (CAG 19-low) showed no significant *HTT* downregulation after transfection with RNAiMAX. Conversely, using a longer ON with low LNA content (CAG 21 low) induced a significant downregulation upon transfection. Examining the actual LNA content in the ONs used (Fig. 2B), we noticed that lowering the LNA content to 31.5% (as in CAG 19-low) abolished the activity. We hypothesized that a minimal LNA content of ∼50% would be required to achieve an anti-gene activity. While not needed for mRNA targeting [50], such a high LNA content might be critical for DNA double-strand invasion/RNA polymerase stalling. Moreover, increased LNA content is expected to increase the intracellular ON stability. We have additionally calculated the melting temperatures (*T*_m_) using the Oligo Analyzer tool from IDT (Fig. 2B and Supplementary Fig. S2). The settings were set to DNA target and the ion and ON concentrations were left at default. We can see a bigger difference in calculated *T*_m_ of CAG 19 ONs than the calculated *T*_m_ of CAG 21 ONs. That, in addition to LNA content, might explain the differences observed in ON activity. We additionally tested CAG 21 ONs with a lower LNA content (∼30%). Although it showed less activity numerically, this effect was not significantly different from other CAG 21 ONs (Supplementary Fig. S2).

These results encouraged us to investigate even longer ONs. To this end, we designed and examined a 45-mer ON (CAG 45 PS) and a construct where three 15-mers were linked together by a three-carbon spacer (CAG 3 × 15 PS) (Table 1). The two longer ONs were fully PS-modified and displayed similar activity when transfected as observed with CAG 15 (∼60% downregulation). However, the CAG 3 × 15 ON with a PO backbone showed a significantly better downregulation compared with the corresponding PO 45-mer (Fig. 2C). It is worth mentioning that upon testing CAG 45 PS ONs with a lower LNA content, we observed a decrease in activity at different doses (Supplementary Fig. S2).

The application of multimers has been reported previously with gapmers by Subramanian *et al.* [51] using PO cleavable linkers. They showed that multimeric structures composed either of the same or different ONs displayed higher efficiency against single or multiple targets *in vivo*. The authors reported that the more stable PS linkers yielded similar results in cellular models but showed less efficacy *in vivo* [51]. Similarly, Bhagat *et al.* [49] reported length-dependent ON activity upon attaching them through their 5′ end and referring to them as gene silencing ONs. Moreover, Brown *et al.* [52] applied the multimeric concept with siRNA. Although these siRNA multimers did not show a significant enhancement compared with the monomeric siRNAs, it allowed multigene silencing by connecting different siRNA units. In addition, the authors emphasized that conjugations can be constructed in any orientation without jeopardizing the overall activity [52].

Recently, Alterman *et al.* [23] reported potent silencing of *HTT* using dimeric siRNA connected through a tetraethylene glycol linker both *in vitro* and *in vivo.*

### Palmitoyl modification of ONs retains anti-gene activity

Modification of ON by conjugation to fatty acids is a common way to enhance the binding to albumin and prolong circulation half-life, thereby increasing the ON uptake [34,35,53]. For example, palmitoyl modifications were reported to increase the half-life of gapmers in blood [36,54], paving the way to reduce the number of PS linkages in the backbone, which have been reported to cause toxicity [55].

To investigate these parameters, we started by analyzing CAG 15 Pal PS (with palmitoylated LNA nucleotides near the 5′ and 3′ ends). It is worth mentioning that the palmitoyl conjugation was introduced on the 2′-amino-LNA nucleotides and not covalently bound to the ON terminals, as usually reported in the literature. Due to technical limitation, we can only insert the palmitoyl modifications on the 2′-amino-LNA-T and 5-methyl-C nucleotides.

We observed a lower level of *HTT* downregulation in the presence of the palmitoyl moiety upon transfection (data not shown). However, the activity upon transfection of palmitoylated CAG 15 ON was equal to non-palmitoylated CAG 15 upon transfection under serum starvation conditions (Supplementary Fig. S3). This might explain the lower activity of palmitoylated ON under standard serum conditions.

### The position of di-palmitoyl modification is critical for anti-gene ON activity

**FIG. 3.**
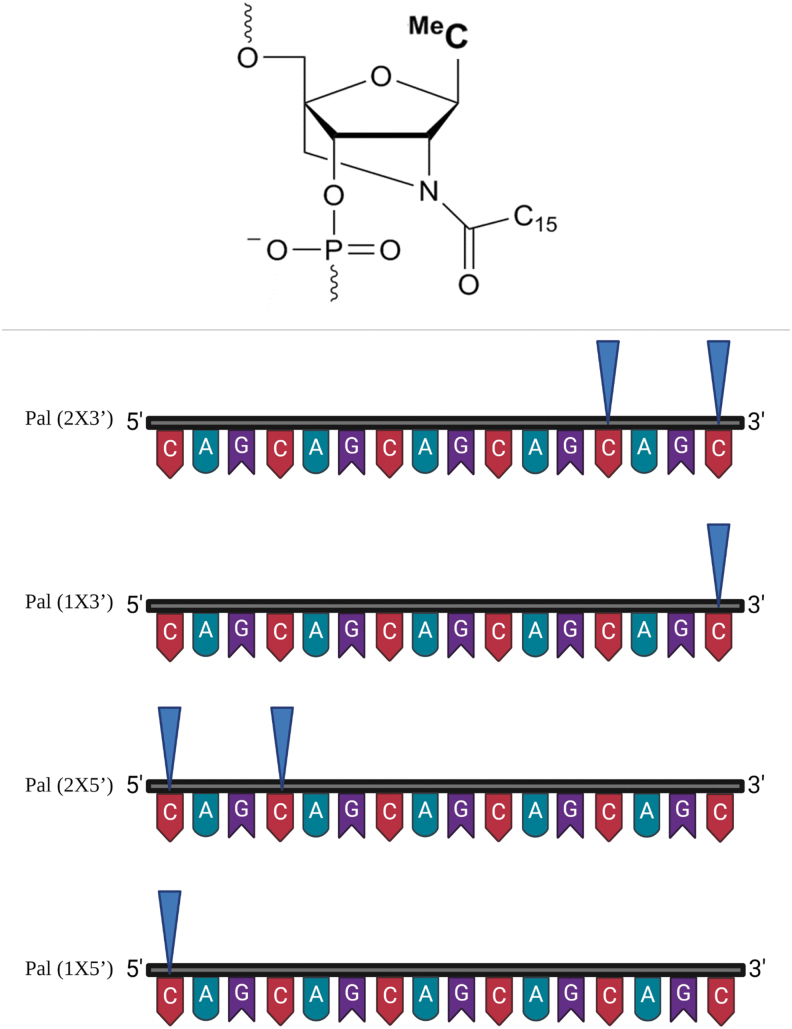
Schematic illustration for the number and positioning of palmitoyl-modified LNA units in CAG 16 ON. *Upper panel:* palmitoylated 2′-amino LNA structure (adapted after permission from John Wiley and Sons^54^). ^Me^C (used in this study) is referring to 5-methylcytosine-1-yl. *Lower panel:* Illustration and naming of the different palmitoyl-conjugated ONs according to the number and the positioning of the palmitoyl groups (*blue triangles*).

To test palmitoyl group modifications at the 3′ and 5′ end, we increased the length of the CAG ON to 16-mers, since palmitoyl modifications of 2′-amino-LNA at present are only available for the pyrimidines, as mentioned before (Table 1 and Fig. 3). We first investigated the effect of having a single palmitoyl-modified LNA at either terminus (3′ or 5′). We later investigated the outcome of having two palmitoylated LNA units, one located in the terminus and the other attached to the adjacent C-nucleotide (Fig. 3). We found that having the two palmitoylated LNA nucleotides at the 3′ end of the ON (Pal 2 × 3′) increased the anti-gene activity compared with the CAG 16 ON or to having two palmitoylated LNA units at the 5′ end of the ON (Pal 2 × 5′).

**FIG. 4.**
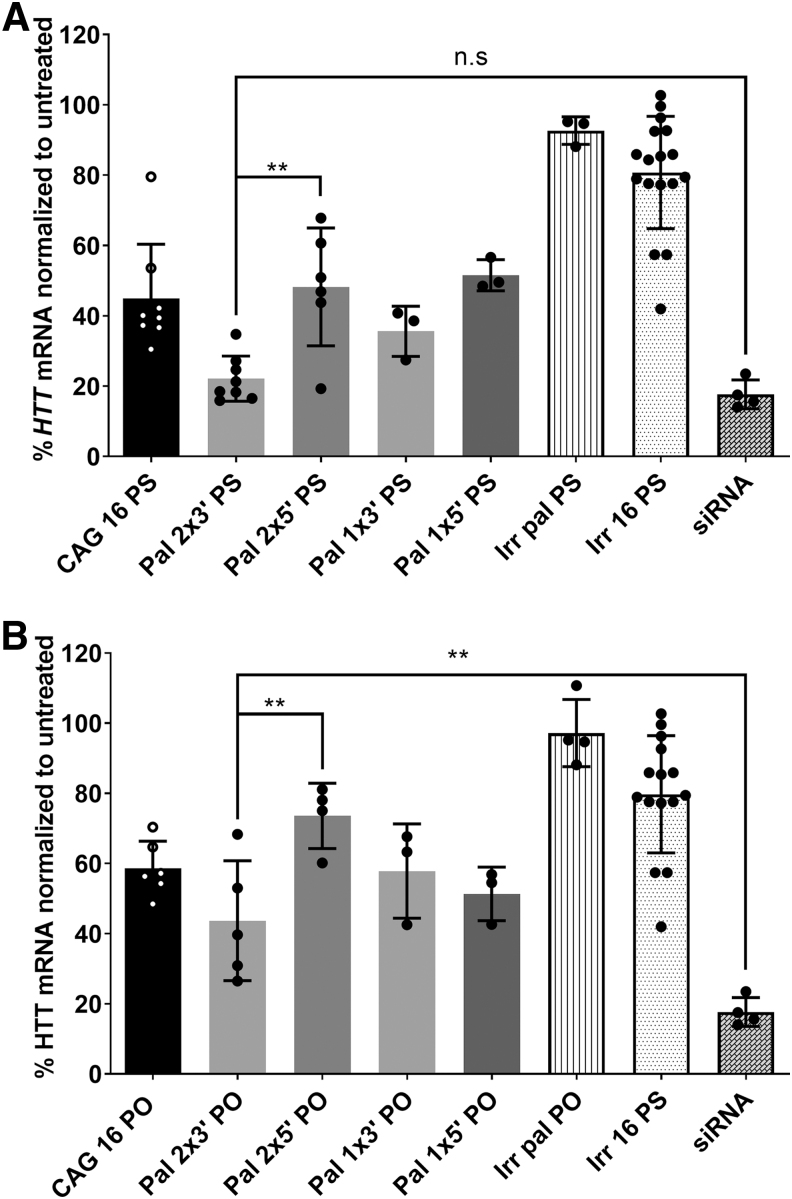
Evaluating the efficacy of different palmitoyl-conjugated anti-gene ONs in downregulating *HTT* mRNA. **(A)** *HTT* mRNA levels 4 days after transfection (100 nM) of various PS backboned 16-mer ONs (sequences in Table 1) into GM04281 human HD fibroblasts carrying 68 repeats on the disease allele. **(B)** *HTT* mRNA levels 4 days after transfection (100 nM) of PO backboned 16-mer ONs (sequences in Table 1) into GM04281 human HD fibroblasts. Irr pal (PS/PO) sequences have the palmitoyl-modified LNA units positioned similarly as in Pal 2 × 3′ ONs. Error bars = SD (*n* ≥ 3), **P ≤ 0.01 (one-way ANOVA, *post hoc* Bonferroni). PO, phosphodiester; PS, phosphorothioate.

The preference of having two palmitoylated LNA units at the 3′ end was evident (Fig. 4A, B) regardless of the chemical modification of the backbone (PS or PO). In fact, the transfection results demonstrated that the efficiency for the palmitoylated PS ON was even similar to the siRNA (Fig. 4A).

Examining published reports did not reveal any preferred positioning for Pal conjugation that has a direct impact on the ON activity. Some studies reported that the 3′ end of ON is the optimal position for conjugation of the sense strand in siRNA and single-stranded ONs [56,57]. Other studies, however, found that attachment to the 3′ end of the ONs greatly decreases the activity [58,59]. In our experimental settings, we tested irrelevant ON sequences with two palmitoylated LNA units at the 3′ end (Irr pal) as a control. This is to investigate whether such a modification could cause downregulation of *HTT* gene expression regardless of the ON sequence. Irrelevant controls with or without palmitoyl modifications did not show any activity (Fig. 4A, B). As an additional control, we tested a different set of primers that targets another region of the gene. We obtained a similar pattern of downregulation that confirms the superiority of “Pal 2 × 3′ PS” over CAG 16 (Supplementary Fig. S4). It is worth mentioning that with the other primer set (targeting a different region of mRNA), the efficacy of ON downregulation appeared to be less (insignificantly in most cases except with the CAG 16 ON).

Wang *et al.* [53] demonstrated that lipid-conjugated ONs accumulate more in cells, and have a faster release from endosomes than unconjugated ONs. The authors also highlighted that the superior uptake and activity of conjugated ONs are related to their protein binding properties [53].

Moreover, lipid conjugation may cause the formation of micellar structures that would influence the uptake efficacy and pathway [60,61] and the endosomal release of ONs [62]. Perhaps the site of conjugation and the number of lipid conjugates would have variable influences on the aforementioned factors. Biscans *et al.* [63] emphasized the importance of the number of conjugated fatty acid units and their effects on siRNA hydrophobicity and aggregation. The authors also highlighted that the valency of the fatty acid conjugate had a significant impact on the hydrophobicity and aggregation of the siRNA, compared with the chemical nature of fatty acid conjugates that had little or no effect.

### Di-palmitoyl-modified ONs show superior anti-gene activity under serum starvation conditions

**FIG. 5.**
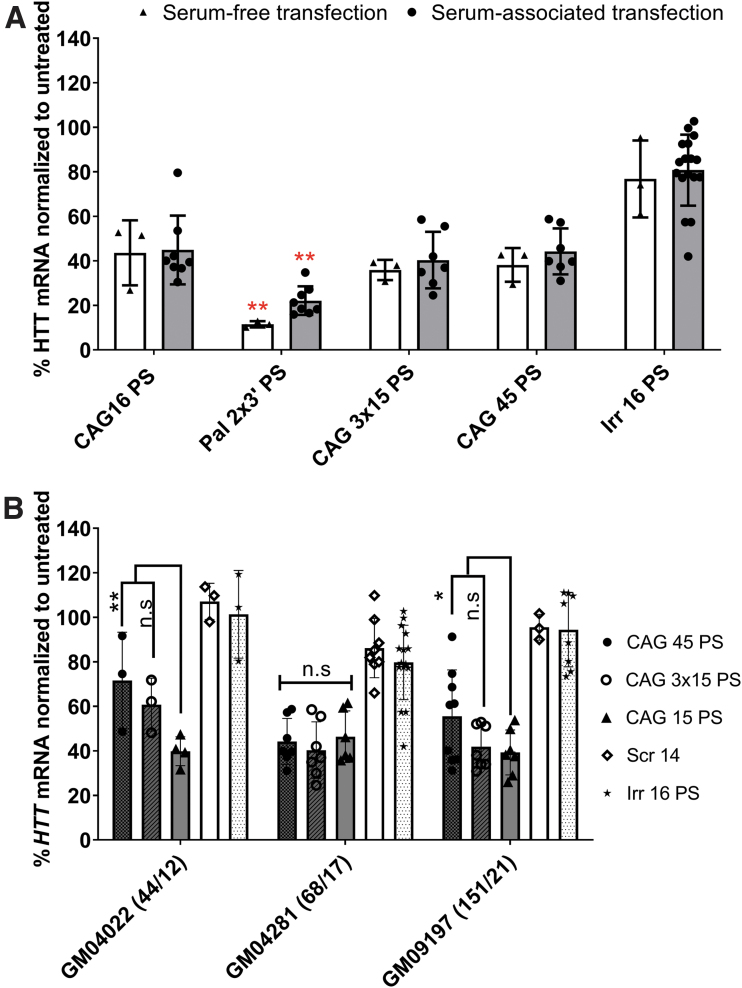
Evaluation of the efficacy of the lead ON candidates under different experimental settings. **(A)** *HTT* mRNA levels 4 days after transfection (involving serum starvation conditions for 4 h) of selected ON candidates with PS backbone (sequences in Table 1) into GM04281 human HD fibroblasts carrying 68 repeats on the disease allele (final concentration 100 nM). **(B)** *HTT* mRNA levels 4 days after transfection (100 nM) of selected long ON candidates with PS backbone (sequences in Table 1) into human HD fibroblasts with different repeat lengths (the numbers between *brackets* refer to the number of repeats of mutant and wild-type *HTT* CAG•CTG trinucleotide repeats). Error bars = SD (*n* ≥ 3), n.s., nonsignificant, *P ≤ 0.05, **P ≤ 0.01 (one or two-way ANOVA, *post hoc* Bonferroni). Red asterick represent the statistical significance of Pal 2 × 3′ PS vs CAG 16 PS effect under serum-free or serum-associated transfection conditions.

Serum is reported to affect the transfection efficacy by various mechanisms [64]. We observed a minimal level of *HTT* downregulation upon transfection using palmitoylated CAG 15 ON under serum conditions (data not shown), contrary to serum starvation conditions (Supplementary Fig. S3). We then tested a selection of optimized ON candidates that were transfected under serum-free conditions for 4 h. followed by replacing the media with a serum containing one for another 92 h. Figure 5A shows that the activity of the palmitoylated ON increased up to 85%, while the activity of the other non-palmitoylated ONs either slightly decreased or remained the same compared with the corresponding treatment under serum conditions (Fig. 4A).

These results may have a predictive value for the performance of the ON at different sites in the body according to the existing protein concentration. Injecting intravenously would allow the palmitoylated ON to bind to protein, circulate for a longer period, and not be easily lost by kidney filtration. However, palmitoylated ON injected intrathecally would be exposed to a low protein concentration, while maintaining enough hydrophobicity to allow better uptake. Prakash *et al.* [35] reported a tissue-specific effect of ON in muscles but not in the liver and kidneys after subcutaneous injection of palmitoylated ON with PS backbone. The same authors reported that both palmitoyl and oleoyl conjugations provided the highest enhancement in ON activity compared with other saturated and unsaturated fatty acid-conjugated ONs.

Similarly, Østergaard *et al.* [34] investigated different lipid conjugations in antisense ONs assayed in nonhuman primates. Palmitoyl- and tocopherol-conjugated ONs showed the highest potency. Nevertheless, palmitoyl conjugation was preferred due to equal efficacy in intravenous and subcutaneous injections [34]. A dose–response relationship was established for selected ON candidates (Supplementary Fig. S5). In the range of doses tested (25–100 nM), the effect of CAG 3 × 15 PS ON remained unchanged.

### Selected anti-gene ON displayed similar results in fibroblasts with different repeat lengths

Fibroblasts carrying either 44/12 or 151/21 CAG•CTG repeats (mutant/wild-type *HTT* alleles) were treated to investigate the efficacy of selected ONs. We chose the longer ONs based on the hypothesis that their efficacy might be affected upon targeting different repeat lengths.

All the tested CAG ONs displayed similar effects as in the GM04281 fibroblasts (Fig. 5B). However, CAG 45 showed a significantly lower activity compared with CAG 15 in fibroblasts with shorter (44/12) and longer (151/21) repeat lengths (GM04022 and GM09197, respectively). The downregulating effect of CAG 3 × 15 ONs was less in GM04022 fibroblasts compared with CAG 15, but the difference was not significant. Therefore, it is reasonable to assume that the number of repeats in GM04022 fibroblasts offers fewer binding sites for such long-ONs (CAG 45 and CAG 3 × 15 ONs) to be sufficiently efficient in downregulating transcription. Yet, it was interesting to see that CAG 3 × 15 was not significantly different from CAG 15 in all repeat lengths tested. This might mean that it can be effective and versatile in early (low somatic expansion) or late (increased length due to somatic expansion) disease stages.

However, it should be noted that compared with cells in the brain, which have acquired a large number of somatically expanded repeats, the difference in lengths among these tested primary fibroblasts is modest.

We also tested if different contents of PS would affect the ON efficacy in fibroblasts carrying variable numbers of repeats. Although we noticed a greater downregulation of *HTT* mRNA in a fully PS-containing CAG 16 compared with a 50% PS, or a full PO CAG 16 ON, these differences were only statistically significant in GM04281 fibroblasts (Supplementary Fig. S6).

### Protein data show variable anti-gene effects based upon ON modifications and backbone

**FIG. 6.**
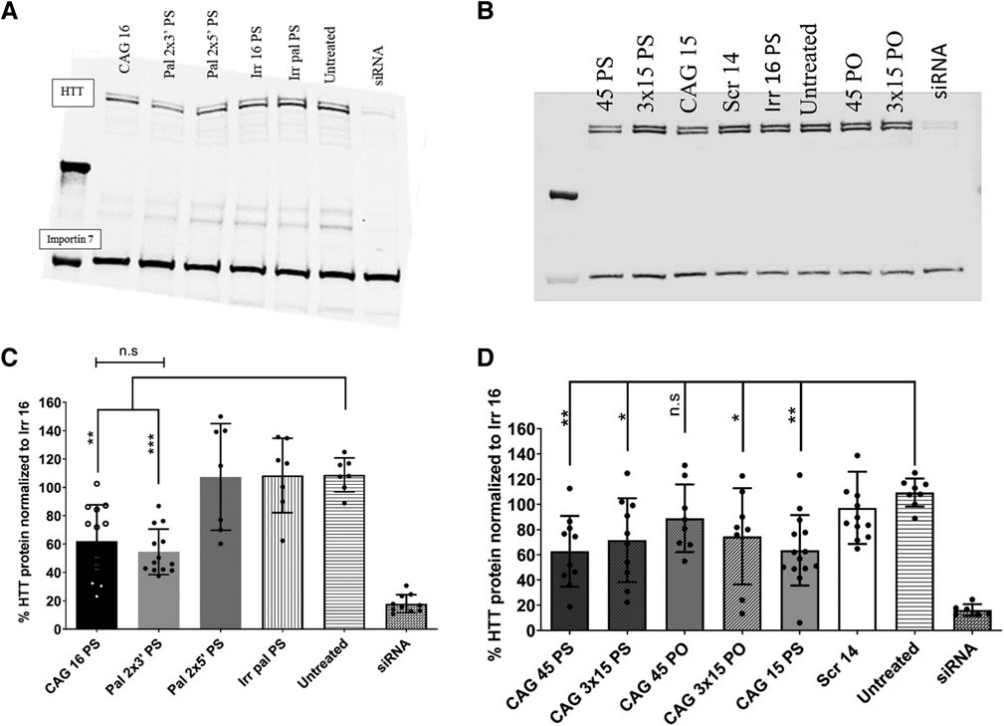
Effects of anti-gene CAG ONs on HTT protein levels in GM04281 human HD fibroblasts. **(A, B)** Representative gels for western blots performed with selected ON candidates. The *top* two bands correspond to HTT protein (upper band mutant; lower band wild-type allele). The band in the *bottom* marks importin 7 protein and used as a loading control. **(C, D)** HTT protein level quantified by ImageJ and normalized to importin 7 after 4 days of transfection of GM04281 human HD fibroblasts carrying 68 repeats on the disease allele (final concentration 100 nM) with different 16 mer CAG ONs with PS backbone and long CAG ON with different backbones, respectively. Error bars = SD (*n* ≥ 3), n.s., nonsignificant, *P ≤ 0.05, **P ≤ 0.01, and ***P ≤ 0.001 (one-way ANOVA, *post hoc* Bonferroni).

After establishing the anti-gene activity on the *HTT* RNA level, for which the quantitative methods are highly sensitive and robust, we investigated the effects of optimal ONs on the HTT protein level. Transfection experiments for the best performing palmitoylated PS ONs demonstrated similar effects on HTT protein and RNA levels. As displayed by Fig. 6A, C, ONs having the two palmitoylated LNA units at the 3′ end of the ON (Pal 2 × 3′) displayed a higher anti-gene activity compared with the ON having two palmitoylated LNA units at the 5′ end (Pal 2 × 5′).

Longer ON (45-mer and 3 × 15-mer) upon transfection demonstrated that CAG 45 PS ON was as active as CAG 15 PS (∼40% downregulation) and slightly more active than the CAG 3 × 15 PS ON (∼30% downregulation) in reducing HTT protein. On the contrary, CAG 45 PO had no effect on the protein level. In contrast, CAG 3 × 15 PO was comparable with CAG 15 PS (∼30% downregulation) and more active than CAG 45 PO (∼20% downregulation) (Fig. 6B, D).

However, we did not see allele selectivity in protein data. In fact, we observed in a previous study that CAG ONs can also downregulate the expression of *HTT* in healthy induced pluripotent stem cells, embryoid bodies, and neural stem cells [39].

Although we see discrepancies between the results of the RT-qPCR data and the protein data, we generally trust the RT-qPCR data because of the sensitivity. The consistency of results observed in siRNA between RT-qPCR data and the protein data can be due to the different mechanisms of action of siRNA. The effect of the siRNA is expected to be faster, affecting the already transcribed pre-mRNA and mRNA simultaneously. The anti-gene ON is expected to prevent the transcription of new pre-mRNA. Therefore, the previously transcribed pre-mRNA and mRNA will not be affected and can be translated into protein.

Finally, the viability of cells was assessed after treatment with selected ONs from our study as well as the control ONs. According to the results, all the tested ONs showed minimal or no toxicity to the tested cells (Supplementary Fig. S7).

### Bioinformatic analysis shows similarity between CAG 16 PS and Pal 2 × 3′ PS

**FIG. 7.**
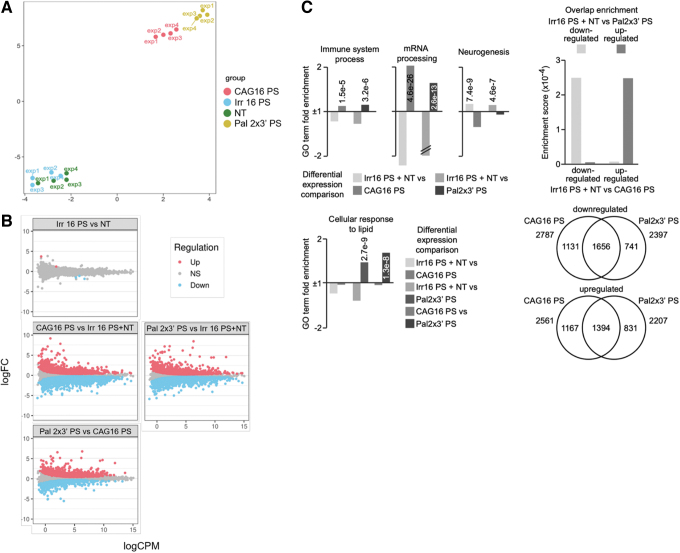
RNAseq analysis of GM04281 human HD fibroblasts carrying 68 repeats on the disease allele 4 days after Lipofectamine RNAiMAX-transfection of PS ON CAG 16 PS, Pal 2 × 3′ PS, both directed against the CAG•CTG tandem repeat, or Irr16 PS, irrelevant control sequence. **(A)** UMAP dimensionality reduction plot of the samples based on the normalized gene counts after filtering the low expressed genes. Exp 1–4 represent four biological replicates. **(B)** The MA [M (log ratio) and A (mean average) scales] plots of selected comparisons illustrating the magnitude of the expression change (*y* axis, log2-fold change) and abundance (*x* axis, log counts per million). *Red* and *blue* colors indicate the significantly up- or downregulated genes, respectively. Nonsignificant genes are represented in *gray*. **(C)** GO term enrichment analysis and differential expression comparison of selected GO terms between the control treated cells, CAG 16 PS, and Pal 2 × 3′ PS (fold enrichment is presented as columns, *P* values are written as numbers, // indicates values that are off the scale). The Venn diagram shows the number of overlapping and nonoverlapping GO terms between CAG 16 PS and Pal 2 × 3′ PS, which are either down- or upregulated. GO, gene ontology; UMAP, Uniform Manifold Approximation and Projection.

Upon confirming the anti-gene activity of tested ONs on HTT mRNA and protein, we utilized RNA sequencing to reveal the broader impact of the ONs on the cell transcriptome. To this end, we selected two targeting ONs, CAG 16 PS and Pal 2 × 3′ PS and one nontargeting ON, Irr 16 PS. A nontreated (NT) control was also included. The cells were transfected under serum conditions as previously described. RT-qPCR validation for the samples was performed before running the sequencing showing comparable results (Supplementary Fig. S8). Dimension reduction technique visualized by Uniform Manifold Approximation and Projection (UMAP) shows that CAG 16 PS and Pal 2 × 3′ PS cluster separately from Irr 16 PS and NT (Fig. 7A). The same clustering pattern was also noticed in the UMAP with removed batch effect (Supplementary Fig. S9). Comparing NT and Irr 16 PS revealed only 5 significantly regulated genes (Fig. 7B).

Therefore, we merged these two groups to be able to remove the outliers based on low RPKM values and increase the power of the consequent analysis with the targeting ONs. The treatment with CAG 16 PS and Pal 2 × 3′ PS led to an increased number of significantly regulated genes (Fig. 7B and Supplementary Tables S1 and S2). The difference between the control ON and targeting ONs can be attributed to the fact that CAG•CTG repeat sequences are more common in coding regions than a random 16 nucleotide-long sequence [65,66].

Since Pal 2 × 3′ PS differs from the CAG 16 PS by the addition of two palmitoylated LNA units at the 3′-end of the ON, we were interested to see if this leads to a different cellular response. The general linear model shows that there are ∼2,000 genes that are either significantly up- or downregulated when comparing these two treatments (Fig. 7B and Supplementary Tables S1 and S2). This is less evident when comparing them separately with NT or Irr 16 PS, which suggests some similarities, but also some differences. We also confirmed that *HTT* is one of the downregulated genes in cells treated with CAG 16 PS and Pal 2 × 3′ PS (Supplementary Fig. S10). More specifically, the GO enrichment analysis of the significant genes (Fig. 7C) indicates that the CAG 16 PS and Pal 2 × 3′ PS equally upregulate 1,656 terms and downregulate 1,394 terms.

However, there are 1,131 and 1,167 terms that are only down- or upregulated by CAG 16 PS. In addition, there are 741 and 831 terms that are only influenced by Pal2 × 3′ PS. Upon more thorough analysis of the terms, we noticed that they similarly influence the immune system process, mRNA processing, and neurogenesis. It has been shown before that ONs induce immune response *in vivo,* and therefore, modulation of this group of genes is not unexpected [67]. Equally can be said for neurogenesis, since HTT is involved in neurodevelopment [11]. However, as expected, CAG 16 PS and Pal 2 × 3′ PS GO enrichment analysis differs in the cellular response to lipids (Fig. 7C). Thus, this is awaited since the palmitoyl moiety is a lipid.

### Both Pal 2 × 3′ PS and CAG 16 PS induce significant up- or downregulation of other repeat containing genes

**FIG. 8.**
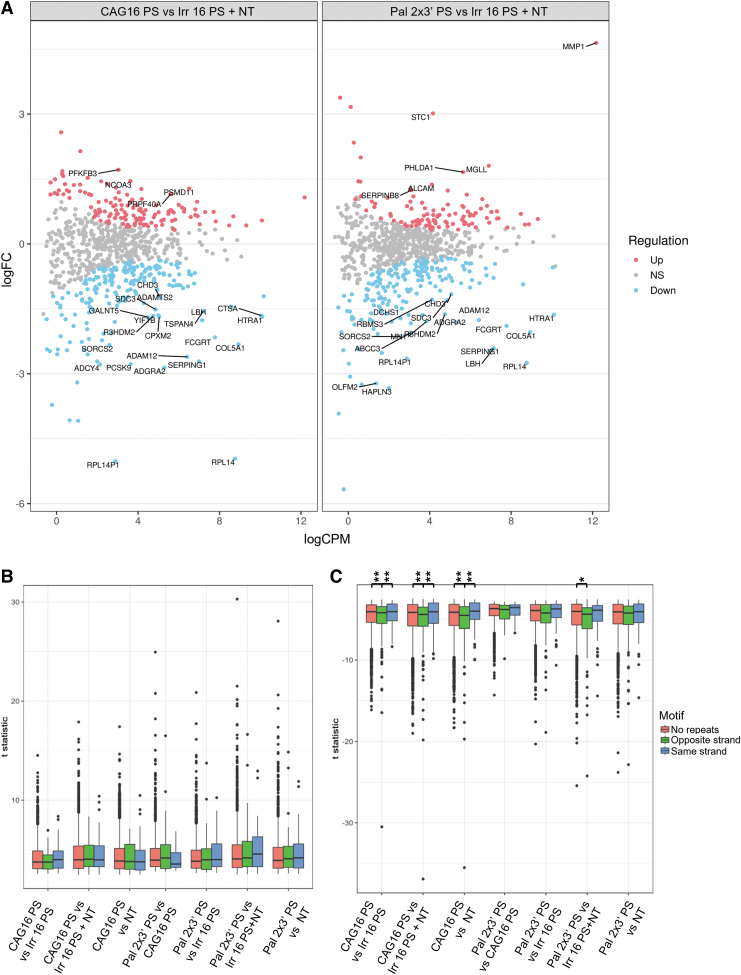
The effect of the CAG 16 PS and Pal 2 × 3′ PS on the expression of genes having at least five CAG·CTG or CTG·CAG repeats. **(A)** The MA plots [M (log ratio) and A (mean average) scales] showing differentially expressed genes comparing the treatment with the targeting ONs to the controls. The MA plots of selected comparisons illustrating the magnitude of the expression change (*y* axis, log2-fold change) and abundance (*x* axis, log counts per million). *Red* and *blue* colors indicate the significantly up- or downregulated genes, respectively. *Top* 25 differentially expressed genes are marked with the gene symbol. **(B, C)** Box plots showing significant up- and downregulated genes in selected comparisons. The genes are divided into three groups—no repeats, CAG repeat on the same strand, or on the opposite strand as the gene orientation. Tukey's HSD test was performed, comparing the *t* statistic of genes when divided into groups depending on the presence of a CAG motif (*P < 0.05, **P < 0.01). HSD, honest significant difference.

*HTT* is not the only gene having a region with multiple CAG•CTG repeats implying that the ONs used in this study can lead to antisense or anti-gene off-targeting. To uncover such potential targets, we used the general linear model to look at the differential expression of all genes having at least five consecutive CAG repeats on either strand. In this analysis, the ON treated samples were compared with the merged NT and Irr 16 PS samples. Although both significantly up- and downregulated genes can be found in both CAG 16 PS- and Pal 2 × 3′ PS-treated samples, 21 and 19 out of 25 most differentially expressed genes are downregulated, respectively. Among these, 13 are the same in both comparisons, with 3 having the CAG repeats on the sense strand and 10 on the antisense strand (Fig. 8A).

To further analyze if the tested ONs have a bias for antisense or anti-gene off-targeting, we used linear model with sample weighting (Voom). Repeat containing genes were divided into two groups—genes with the five CAG repeats on the sense strand (same orientation as the gene) or on the antisense strand (opposite orientation as the gene). In CAG 16 PS-treated cells compared with controls, there is a significant difference between downregulated genes having the repeats on the antisense strand and genes having no or sense strand repeats, suggesting bias toward antisense off-targeting (Fig. 8C). This might be due to the fact that ONs might reach the RNA species in the cytoplasm and nucleus before the chromosomal DNA [68]. However, this needs further analysis since significant difference in Pal 2 × 3′ PS-treated cells can only be observed when compared with combined NT and Irr16 PS samples.

Finally, neither Pal 2 × 3′ PS nor CAG 16 PS induced any significant upregulation of these repeat containing genes (Fig. 8B). A limitation we have in the RNAseq analysis of *HTT* gene is the inability to determine from which strand the read/transcript originates from, because the RNAseq library used is unstranded, and hence, the reads align equally to both strands.

## Conclusion

In this report, we investigated the activity of various anti-gene ONs targeting expanded CAG•CTG DNA repeats in the *HTT* gene. The ONs were LNA/DNA mixmers with PS or PO backbones. The LNA content but not ON length had an impact on activity. Palmitoylated LNA nucleotides had a position-dependent influence over ON activity. The inclusion of two lipophilic units near the 3′end of the ON (Pal 2 × 3′ PS) was most compelling. Bioinformatic analysis showed similarity between CAG 16 PS and Pal 2 × 3′ PS ONs, yet only the latter showed a significant difference in the cellular response to the lipid GO enrichment term. In addition, we highlighted the potential application of multimeric ON sequences. These longer multimeric ONs could maintain the downregulation activity to the same level as their parent shorter ON sequences.

While most of the transfected anti-gene ONs showed simultaneous downregulation of HTT RNA and protein, further efforts are needed to explain the discrepancies between levels of HTT RNA and protein upon transfection of various anti-gene ONs. Future perspectives might also include investigation of anti-gene ONs designed for other TNR diseases as well as the effect of LNA content on their efficacy.

## Supplementary Material

Supplemental data

Supplemental data

Supplemental data

Supplemental data

Supplemental data

Supplemental data

Supplemental data

Supplemental data

Supplemental data

Supplemental data

Supplemental data

Supplemental data
