# Supplementary material for: Chemical Modifications and Design Influence the Potency of Huntingtin Anti-Gene Oligonucleotides
Source: Nucleic Acid Ther. 2023 Mar 30;33(2):117–31. doi: 10.1089/nat.2022.0046 (PMC10066784; doi:10.1089/nat.2022.0046)
Supplement: Supplemental data [file Suppl_FigS1.docx]

**
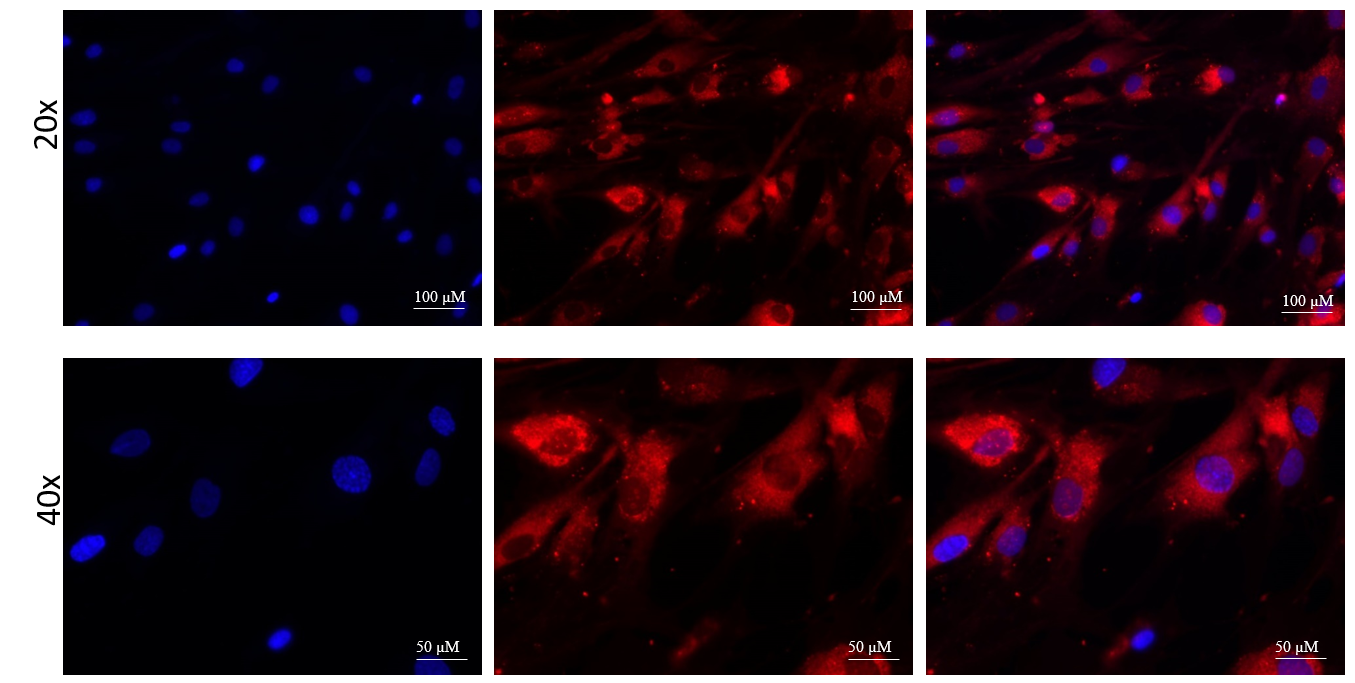
Supplementary figures and legends:
Supplementary Figure S1. Uptake pattern of CAG 19 ON upon transfection.**

GM04281 cells (carrying 68 repeats on the disease allele) were treated with the Cy3 labelled CAG 19 ON for 4 days. The cells were then washed and stained for 30 minutes with Hoechst 33342 (Thermofisher^®^) to visualize the nucleus. This was followed by washing, replacing with fresh DMEM^®^ medium and imaging with fluorescence microscope (Olympus IX81). The images shown are with magnification 20 and 40x, Scale bar = 100 and 50 µm, respectively).
