# Supplementary material for: Chemical Modifications and Design Influence the Potency of Huntingtin Anti-Gene Oligonucleotides
Source: Nucleic Acid Ther. 2023 Mar 30;33(2):117–31. doi: 10.1089/nat.2022.0046 (PMC10066784; doi:10.1089/nat.2022.0046)
Supplement: Supplemental data [file Suppl_FigS2.docx]

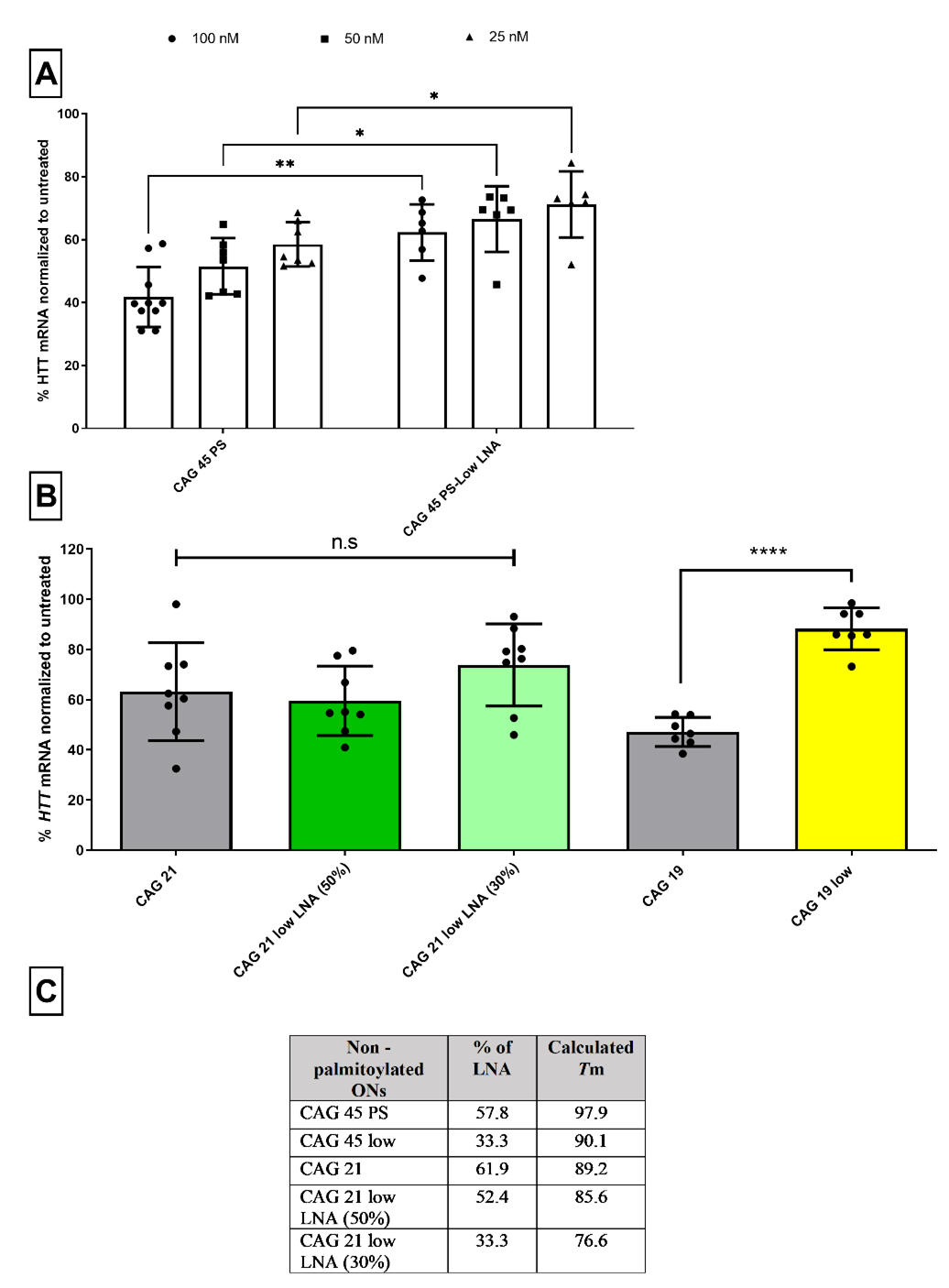


**Supplementary Figure S2. Effect of LNA content on the efficiency of ONs to down-regulate *HTT* mRNA.** (**A**) *HTT* mRNA levels 4 days after transfection of GM04281 human HD fibroblasts carrying 68 repeats on the disease allele with ON (**B**) *HTT* mRNA levels 4 days after transfection of GM04281 human HD fibroblasts with long ONs having different backbone (100 nM) (**C**) Percentage of LNA and calculated *T*m in the different ON used. Error bars = SD (n ≥ 3), n.s.: non-significant, * p ≤ 0.05, ** p ≤ 0.01, **** p ≤ 0.0001 (one- or two-way ANOVA, *post hoc* Bonferroni)
