# Supplementary material for: Chemical Modifications and Design Influence the Potency of Huntingtin Anti-Gene Oligonucleotides
Source: Nucleic Acid Ther. 2023 Mar 30;33(2):117–31. doi: 10.1089/nat.2022.0046 (PMC10066784; doi:10.1089/nat.2022.0046)
Supplement: Supplemental data [file Suppl_FigS3.docx]

**
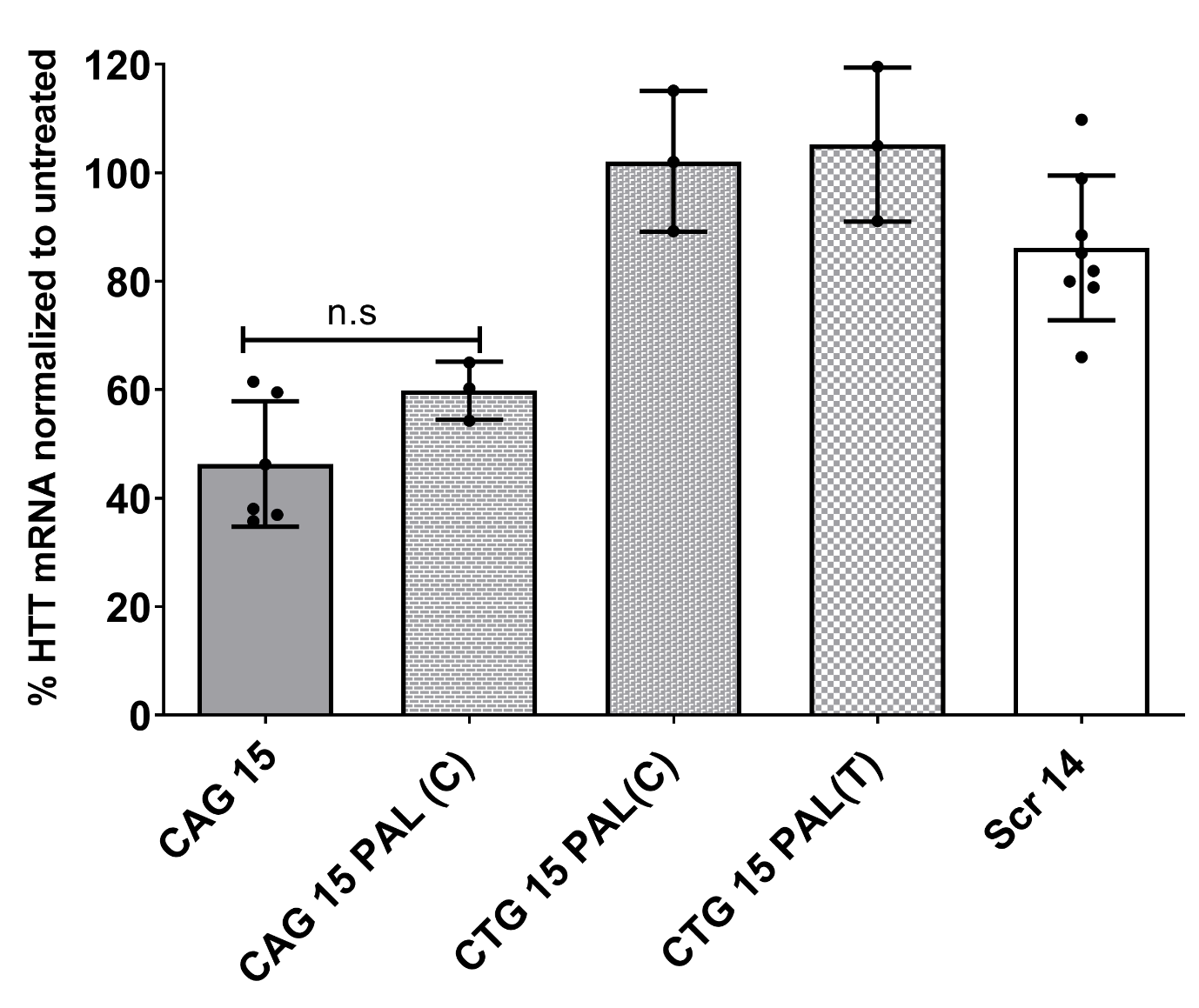
**

**Supplementary Figure S3.** **Effect of Palmitoylation on the efficiency of CAG ONs to downregulate HTT mRNA.**

*HTT* mRNA levels 4 days after transfection (involving serum starvation conditions for 4 h) of 15mer ON with and without palmitoylation (100 nM) into GM04281 human HD fibroblasts carrying 68 repeats on the disease allele. CTG palmitoylated ONs were used as controls where (C) and (T) represent the type of the LNA nucleotide carrying the palmitoyl modification (Table 1). Error bars = SD (n ≥ 3), n.s.: non-significant. (one-way ANOVA, *post hoc* Bonferroni)
