# Supplementary material for: Chemical Modifications and Design Influence the Potency of Huntingtin Anti-Gene Oligonucleotides
Source: Nucleic Acid Ther. 2023 Mar 30;33(2):117–31. doi: 10.1089/nat.2022.0046 (PMC10066784; doi:10.1089/nat.2022.0046)
Supplement: Supplemental data [file Suppl_FigS4.docx]

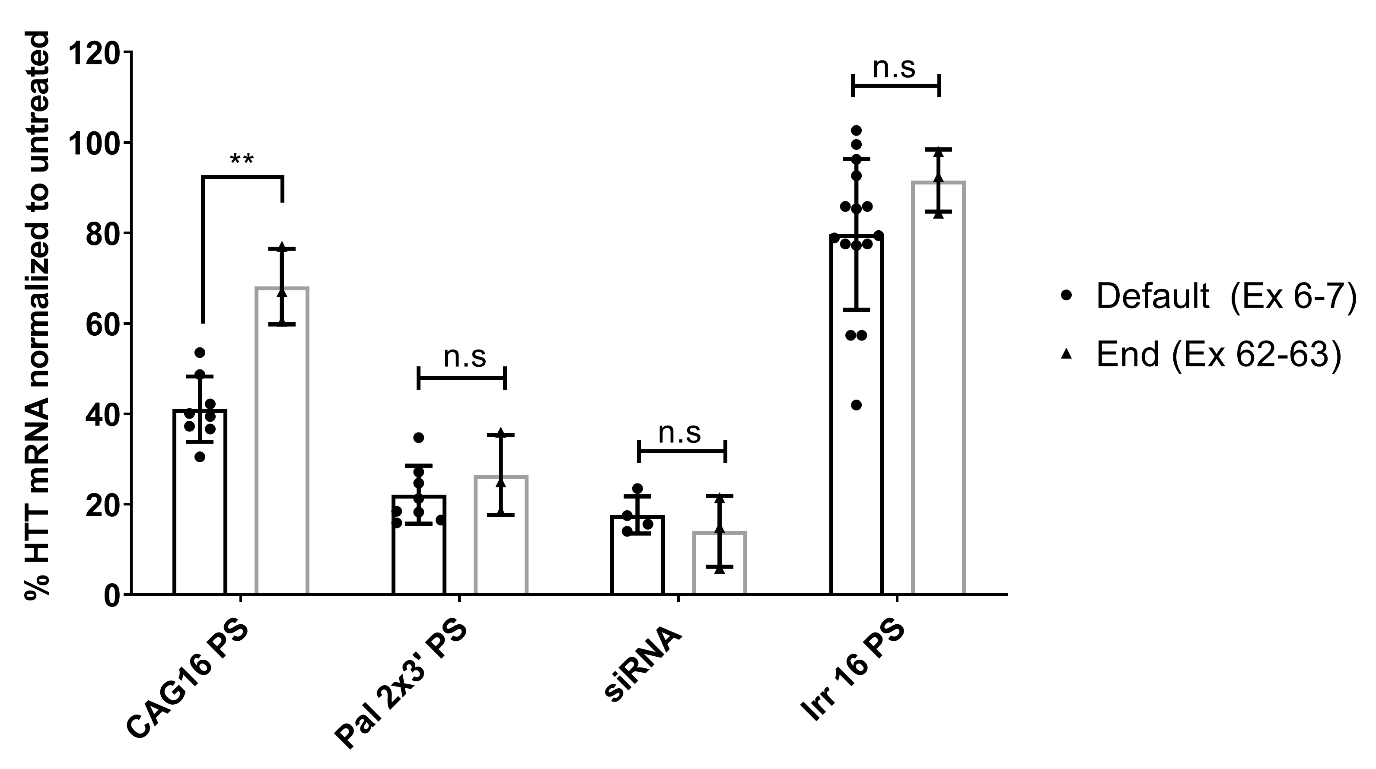


**Supplementary Figure S4. Evaluation of CAG ONs downregulation effect on *HTT* mRNA using different primer sets.**

*HTT* mRNA levels 4 days after transfection (100 nM) of selected ONs into GM04281 human HD fibroblasts carrying 68 repeats on the disease allele. Primer sets targeting the end region (Ex 62-63) of the mRNA were evaluated and compared to the standard primer set (Ex 6-7) used throughout the study. Sequences of primers targeting end region are HTT FW: acctcattgagaactatcc, HTT REV: gcacagtggtaaatgatg, HPRT FW: gatccattcctatgactgta, HPRT REV: tctccaccaattacttttatg. Sequences for taqman probes used are HTT probe: [JOE] actcctcacttccagacagcat [BHQ1], HPRT probe: [6FAM]cctgttgactggtcattacaatagctc [BHQ1].

Error bars = SD (n ≥ 3). n.s.: non-significant, * p ≤ 0.05, ** p ≤ 0.01 (two-way ANOVA, *post hoc* Bonferroni)
