# Supplementary material for: Chemical Modifications and Design Influence the Potency of Huntingtin Anti-Gene Oligonucleotides
Source: Nucleic Acid Ther. 2023 Mar 30;33(2):117–31. doi: 10.1089/nat.2022.0046 (PMC10066784; doi:10.1089/nat.2022.0046)
Supplement: Supplemental data [file Suppl_FigS5.docx]

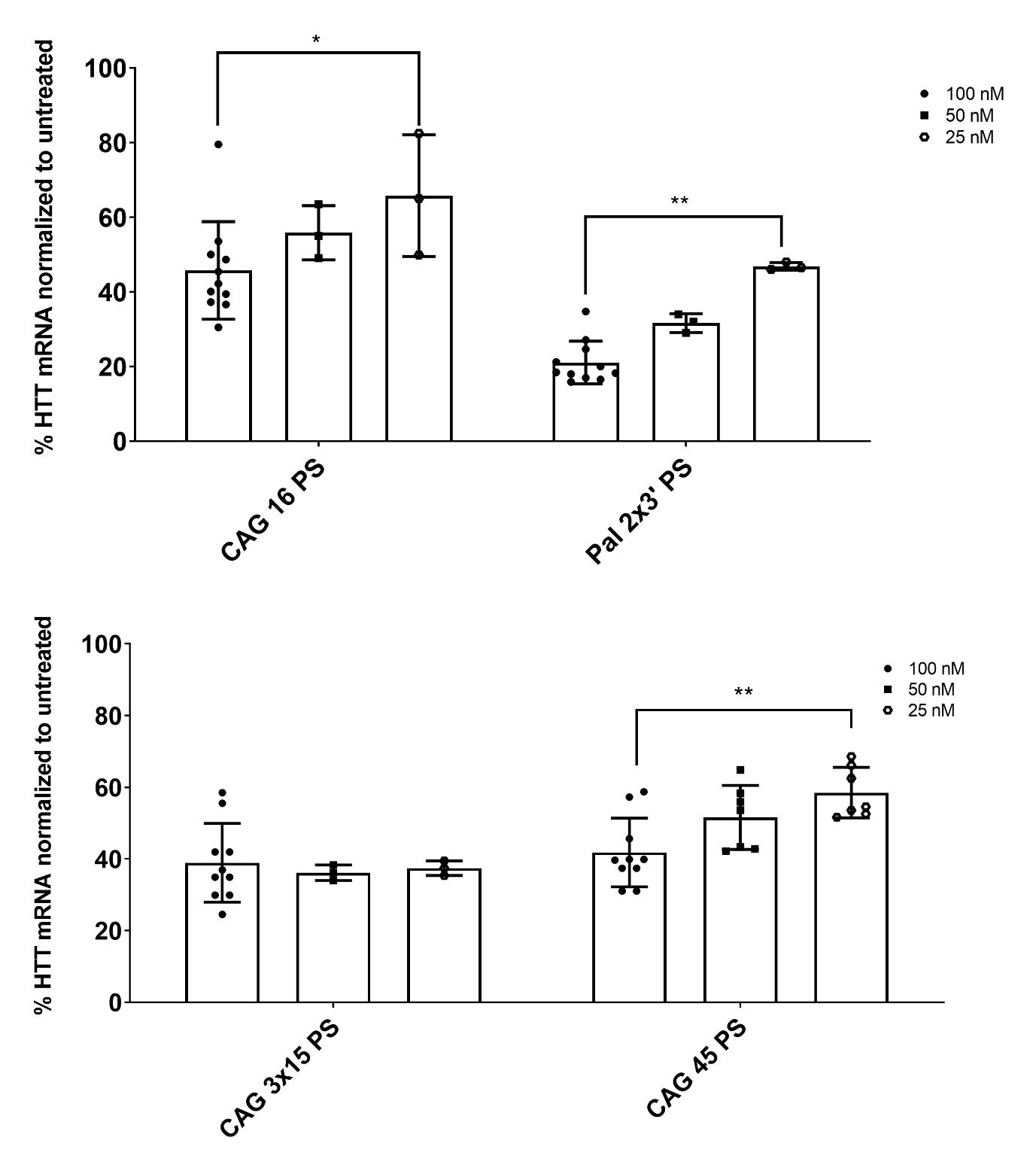


**Supplementary Figure S5. A dose response correlation using 3 different doses (25, 50,100 nM) of selected ani-gene ON.** Upper panel includes CAG 16 PS and Pal 2x3’ PS, the lower panel has CAG 3x15 PS and CAG 45 PS. Error bars = SD (n ≥ 3), n.s.: non-significant, * p ≤ 0.05, ** p ≤ 0.01 (two-way ANOVA, *post hoc* Bonferroni).
