# Supplementary material for: Chemical Modifications and Design Influence the Potency of Huntingtin Anti-Gene Oligonucleotides
Source: Nucleic Acid Ther. 2023 Mar 30;33(2):117–31. doi: 10.1089/nat.2022.0046 (PMC10066784; doi:10.1089/nat.2022.0046)
Supplement: Supplemental data [file Suppl_FigS6.docx]

**
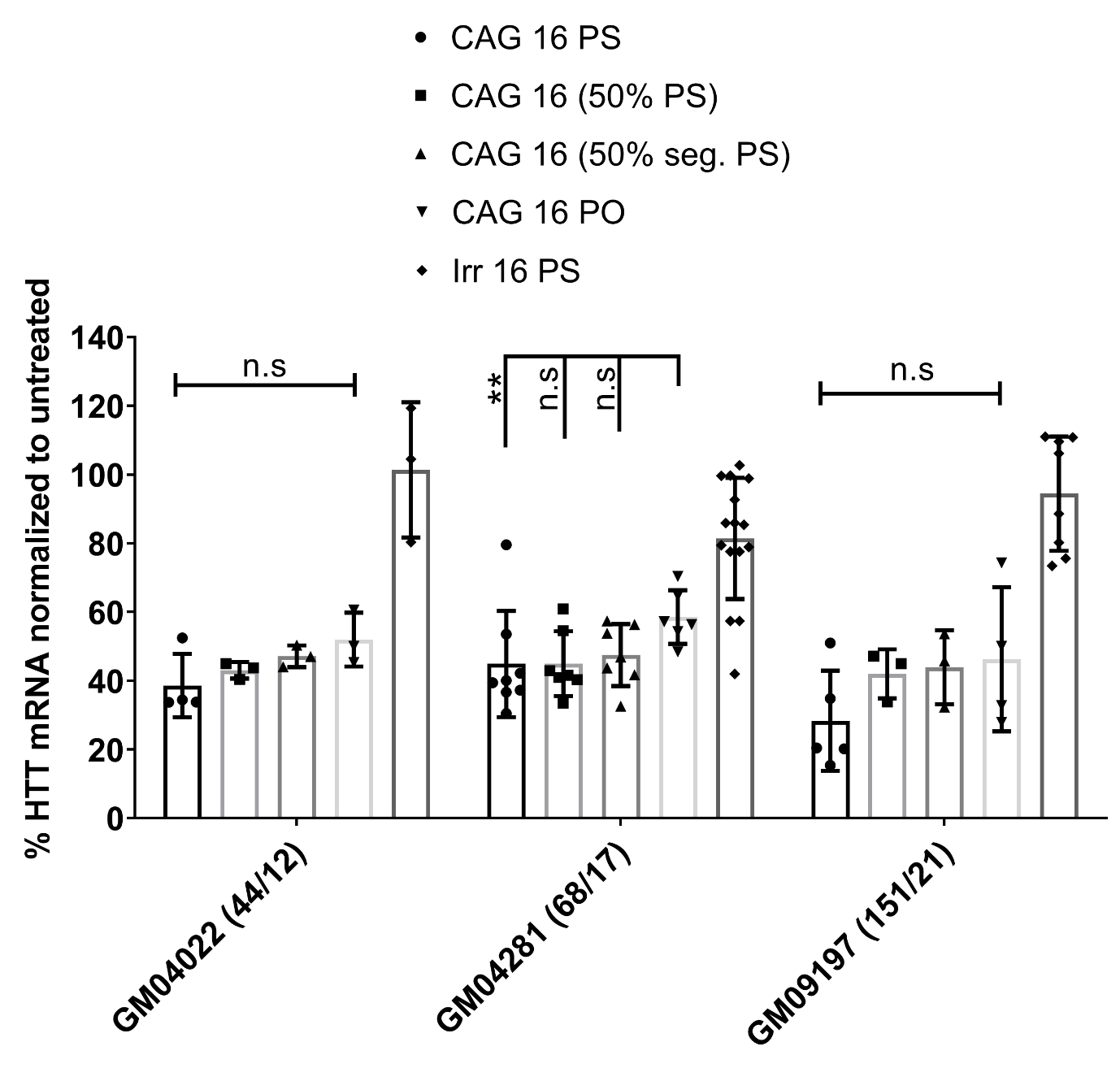
**

**Supplementary Figure S6. Evaluation of phosphorothioate (PS) content and distribution on the efficiency of CAG ONs to downregulate *HTT* mRNA.**

*HTT* mRNA levels 4 days after transfection (100 nM) of selected ON candidates (Sequences in Table 1) into human HD fibroblasts with different repeat lengths (the numbers between brackets refer to the ratio between the repeat length of mutant to wild-type *HTT* CAG•CTG trinucleotide repeats). ON candidates have different distribution of phosphorothioate (PS) and phosphodiester (PO) backbones (PS = 100% PS, 50% PS = alternate mix of PS and PO nucleotides, 50% seg. PS = segregated PS - first half of the sequence (starting from the 5’) is PO and the second is PS). Error bars = SD (n ≥ 3), n.s.: non-significant, and ** p ≤ 0.01 (two-way ANOVA, *post hoc* Bonferroni).
