# Supplementary material for: Chemical Modifications and Design Influence the Potency of Huntingtin Anti-Gene Oligonucleotides
Source: Nucleic Acid Ther. 2023 Mar 30;33(2):117–31. doi: 10.1089/nat.2022.0046 (PMC10066784; doi:10.1089/nat.2022.0046)
Supplement: Supplemental data [file Suppl_FigS7.docx]

**
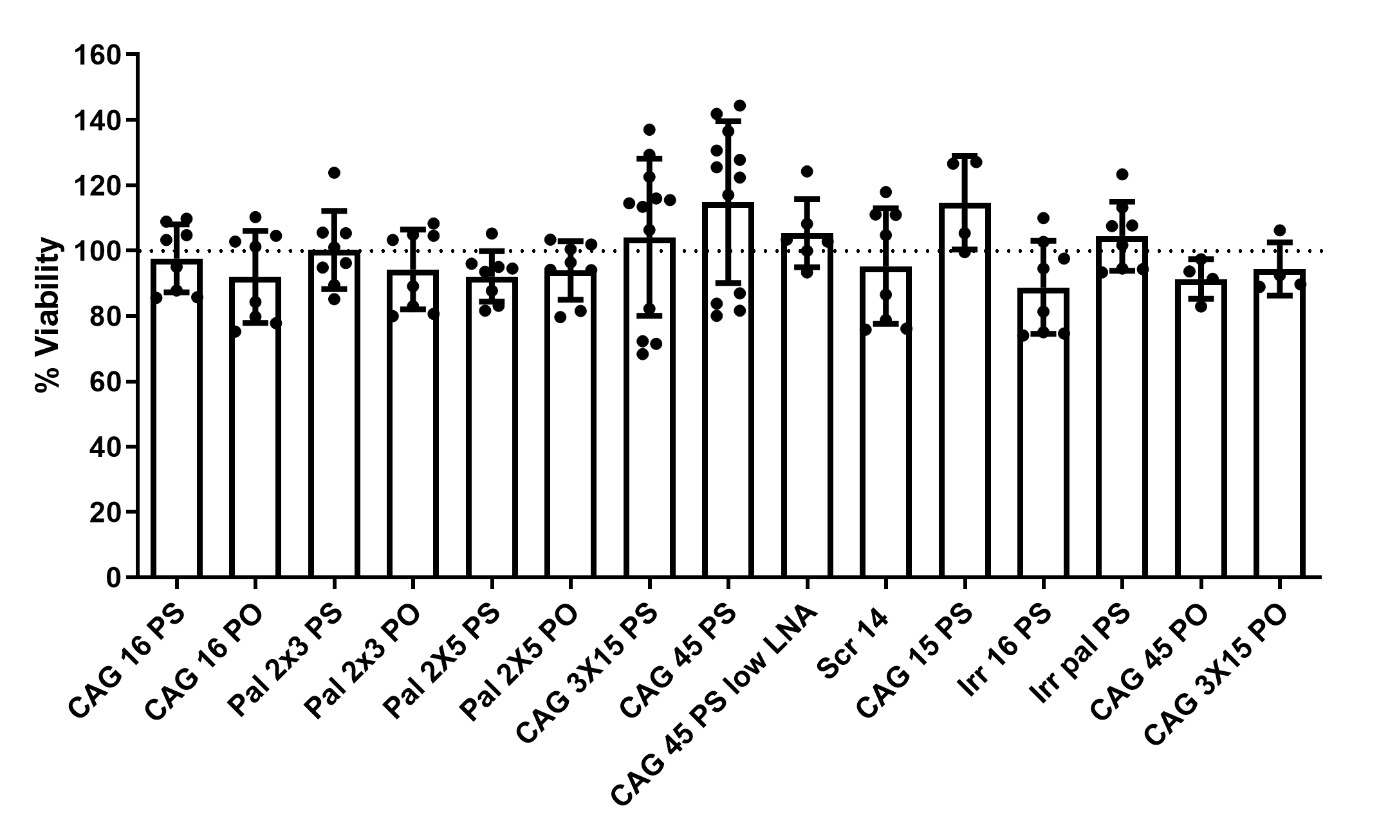
**

**Supplementary Figure S7. Evaluation of the cytotoxicity of the oligonucleotides used in the study.**

Viability normalized to untreated cells presented in percentage (%) (GM04281 human HD fibroblasts, carrying 68 repeats on the disease allele) after transfection with selected ON candidates (Sequences in Table 1). Error bars = SD (n ≥ 3), n.s.: non-significant, * p ≤ 0.05, and ** p ≤ 0.01, *** p ≤ 0.001, (one-way ANOVA, *post hoc* Bonferroni)
