# Supplementary material for: Chemical Modifications and Design Influence the Potency of Huntingtin Anti-Gene Oligonucleotides
Source: Nucleic Acid Ther. 2023 Mar 30;33(2):117–31. doi: 10.1089/nat.2022.0046 (PMC10066784; doi:10.1089/nat.2022.0046)
Supplement: Supplemental data [file Suppl_FigS8.docx]

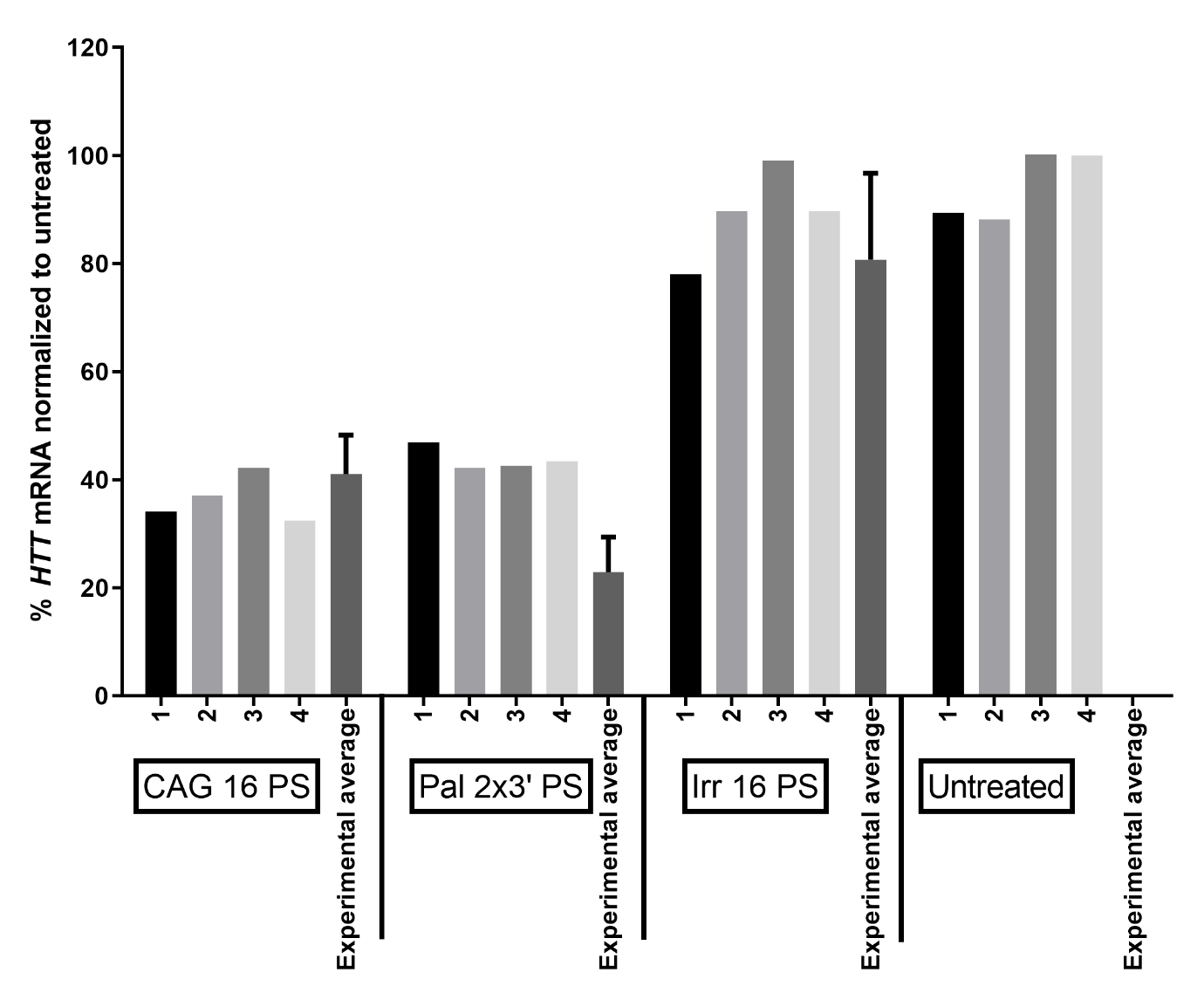


**Supplementary Figure S8. Validation of Sequencing results.** Levels of Expression of the *HTT* mRNA in samples prepared for RNA sequencing analysis.
