# Supplementary material for: Chemical Modifications and Design Influence the Potency of Huntingtin Anti-Gene Oligonucleotides
Source: Nucleic Acid Ther. 2023 Mar 30;33(2):117–31. doi: 10.1089/nat.2022.0046 (PMC10066784; doi:10.1089/nat.2022.0046)
Supplement: Supplemental data [file Suppl_FigS9.docx]

**
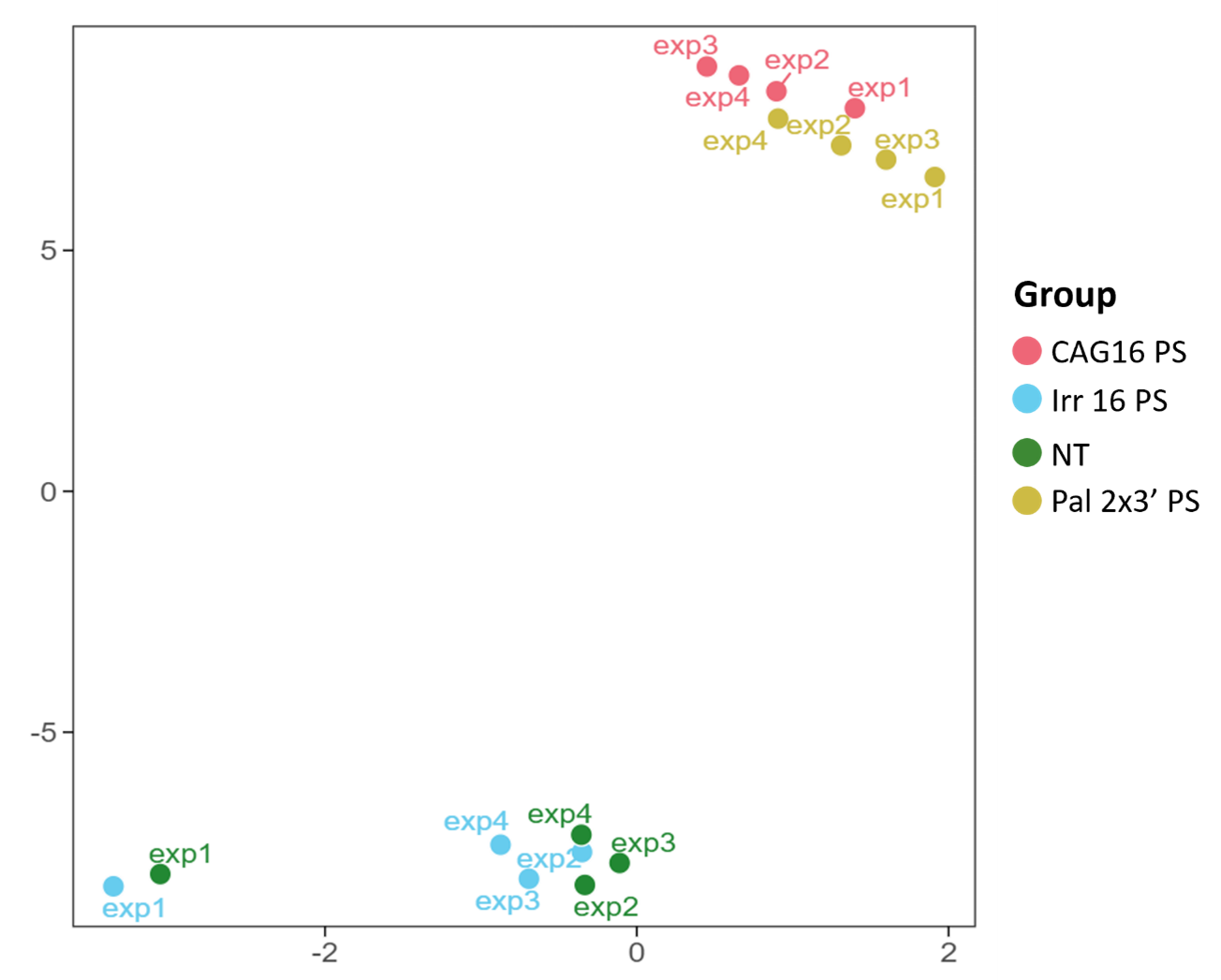
**

**Supplementary Figure S9. rmB UMAP (removed batch effect Uniform Manifold Approximation and Projection) dimensionality reduction plot of the samples based on the normalized gene counts after filtering the low expressed genes.** Exp 1-4 represent four biological replicates.
