# Supplementary material for: Chemical Modifications and Design Influence the Potency of Huntingtin Anti-Gene Oligonucleotides
Source: Nucleic Acid Ther. 2023 Mar 30;33(2):117–31. doi: 10.1089/nat.2022.0046 (PMC10066784; doi:10.1089/nat.2022.0046)
Supplement: Supplemental data [file Suppl_TableS1.docx]

**Supplementary Table 1**: The number of significant genes obtained in each specified comparison using the general linear models (GLM). The table shows genes found significant after p value adjustment for multiple hypothesis testing (FDR). Genes were termed significant if the adjusted p value was under 0.05.

| **Comparison** | **Up regulated** | **Down regulated** | **Non-** **significant** |
| --- | --- | --- | --- |
| Irr 16 PS vs NT | 2 | 3 | 13080 |
| CAG16 PS vs NT | 2304 | 2588 | 8193 |
| CAG16 PS vs Irr 16 PS | 1960 | 2257 | 8868 |
| CAG16 PS vs_Irr 16 + NT | 2561 | 2787 | 7737 |
| Pal 2x3’ PS vs NT | 1823 | 2088 | 9174 |
| Pal 2x3’ PS vs Irr 16 PS | 1641 | 1871 | 9573 |
| Pal 2x3’ PS vs Irr 16 PS + NT | 2207 | 2397 | 8481 |
| Pal 2x3’ PS vs CAG16 PS | 1171 | 970 | 10944 |
