# Supplementary material for: Chemical Modifications and Design Influence the Potency of Huntingtin Anti-Gene Oligonucleotides
Source: Nucleic Acid Ther. 2023 Mar 30;33(2):117–31. doi: 10.1089/nat.2022.0046 (PMC10066784; doi:10.1089/nat.2022.0046)
Supplement: Supplemental data [file Suppl_TableS2.docx]

**Supplementary Table 2**: The number of significant genes obtained in each specified comparison using the general linear models (GLM) after removal of outliers due to low RPKM. The table shows genes found significant after p value adjustment for multiple hypothesis testing (FDR). Genes were termed significant if the adjusted p value was under 0.05.

| **Comparison** | **Up regulated** | **Down regulated** | **Non-** **significant** |
| --- | --- | --- | --- |
| Irr 16 PS vs NT | 1 | 1 | 12729 |
| CAG16 PS vs_Irr 16 + NT | 2474 | 2694 | 7536 |
| Pal 2x3’ PS vs Irr 16 PS + NT | 2192 | 2322 | 8217 |
| Pal 2x3’ PS vs CAG16 PS | 1380 | 1103 | 9573 |
