# Supplementary material for: Chemical Modifications and Design Influence the Potency of Huntingtin Anti-Gene Oligonucleotides
Source: Nucleic Acid Ther. 2023 Mar 30;33(2):117–31. doi: 10.1089/nat.2022.0046 (PMC10066784; doi:10.1089/nat.2022.0046)
Supplement: Supplemental data [file Suppl_FigS10.docx]

**
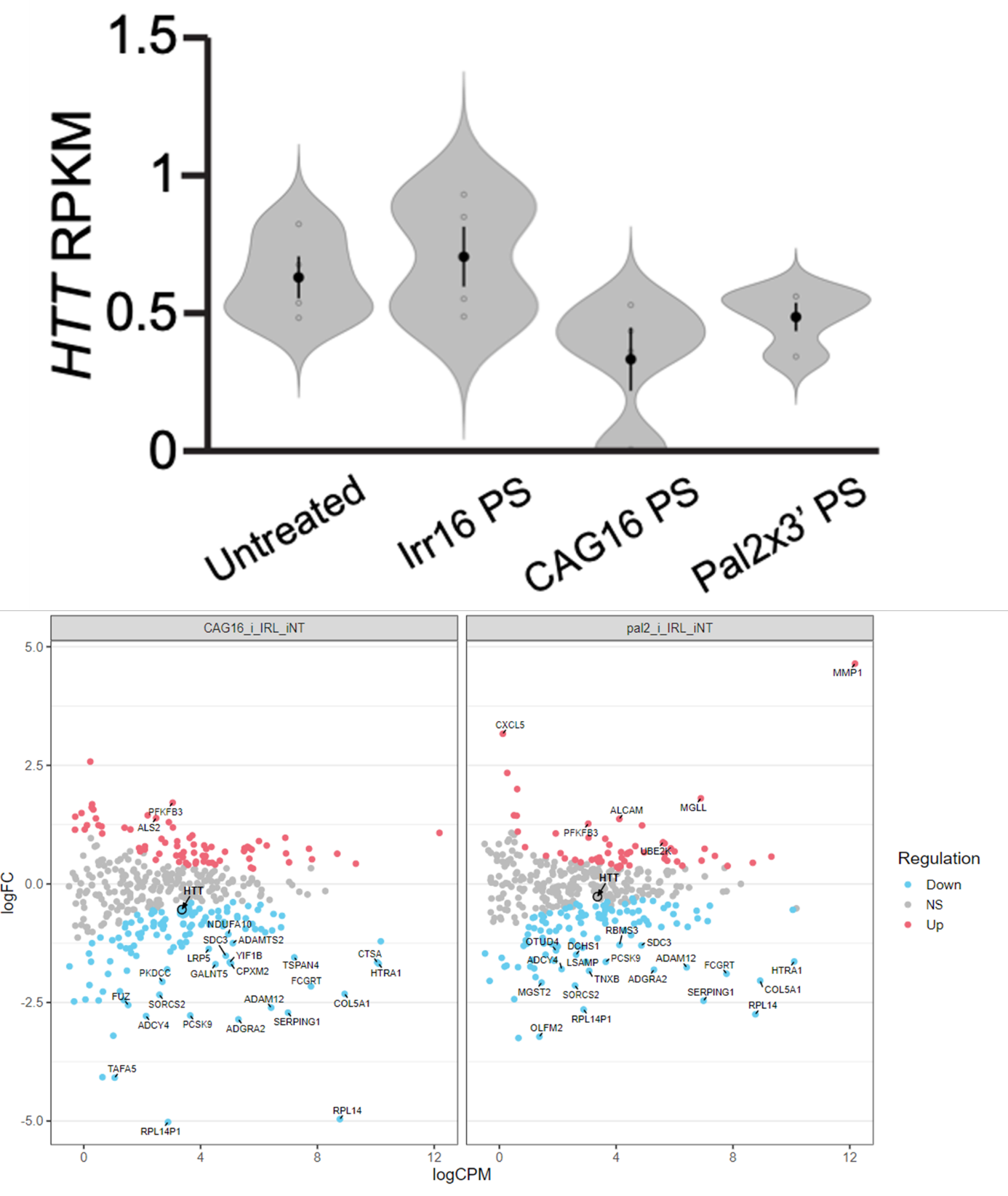

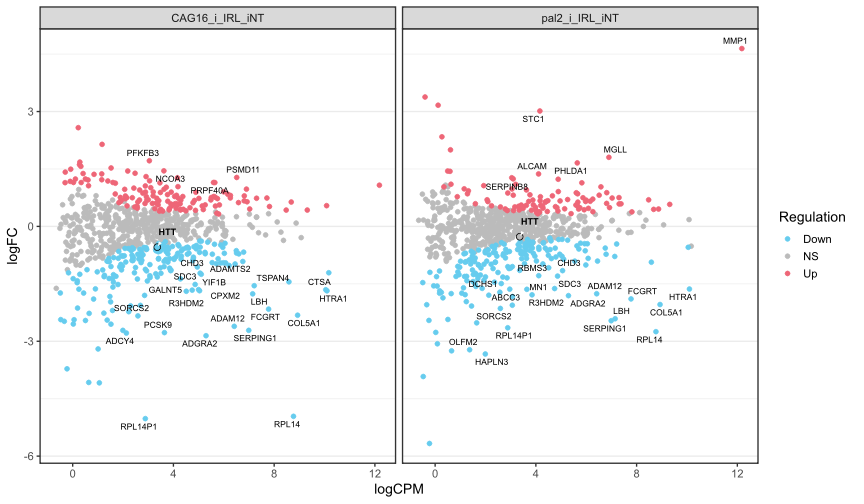
**

**Supplementary Figure S10. Expression of the *HTT* mRNA as determined by RNA sequencing analysis.** CAG16 PS, Pal 2x3’ PS and Irr16 PS were transfected using Lipofectamine RNAiMAX into GM04281 human HD fibroblasts carrying 68 repeats on the disease allele. Upper panel: The violin plot shows the rpkm values of four biological experiments. Lower panel: show location of *HTT* gene on the MA plots (M (log ratio) and A

(mean average) scales). MA plots are showing differentially expressed genes comparing the treatment with the targeting ONs to the controls. The MA plots of selected comparisons illustrating the magnitude of the expression change (y axis, log2 fold change) and abundance (x axis, log counts per million). Red and blue color indicate the significantly up- or down-regulated genes, respectively).
